# Integrated single cell and spatial transcriptomics reveal autoreactive differentiated B cells in joints of early rheumatoid arthritis

**DOI:** 10.1038/s41598-022-15293-5

**Published:** 2022-07-13

**Authors:** Uta Hardt, Konstantin Carlberg, Erik af Klint, Peter Sahlström, Ludvig Larsson, Annika van Vollenhoven, Susana Hernandez Machado, Lena Israelsson, Khaled Amara, Karine Chemin, Marina Korotkova, Gunilla B. Karlsson Hedestam, Anca I. Catrina, Sarah A. Teichmann, Patrik L. Ståhl, Vivianne Malmström

**Affiliations:** 1 Division of Rheumatology, Department of Medicine Solna, Center for Molecular Medicine, Karolinska Institutet, Stockholm, Sweden; 2 Karolinska University Hospital, Stockholm, Sweden; 3 Department of Microbiology, Tumor and Cell Biology, Karolinska Institutet, Stockholm, Sweden; 4 Department of Gene Technology, Science for Life Laboratory, KTH Royal Institute of Technology, Stockholm, Sweden; 5 Wellcome Sanger Institute, Wellcome Genome Campus, Hinxton, Cambridge, CB10 1SA UK

**Keywords:** Biotechnology, Rheumatology

## Abstract

B cells play a significant role in established Rheumatoid Arthritis (RA). However, it is unclear to what extent differentiated B cells are present in joint tissue already at the onset of disease. Here, we studied synovial biopsies (n = 8) captured from untreated patients at time of diagnosis. 3414 index-sorted B cells underwent RNA sequencing and paired tissue pieces were subjected to spatial transcriptomics (n = 4). We performed extensive bioinformatics analyses to dissect the local B cell composition. Select plasma cell immunoglobulin sequences were expressed as monoclonal antibodies and tested by ELISA. Memory and plasma cells were found irrespective of autoantibody status of the patients. Double negative memory B cells were prominent, but did not display a distinct transcriptional profile. The tissue architecture implicate both local B cell maturation via T cell help and plasma cell survival niches with a strong CXCL12–CXCR4 axis. The immunoglobulin sequence analyses revealed clonality between the memory B and plasma cell pools further supporting local maturation. One of the plasma cell-derived antibodies displayed citrulline autoreactivity, demonstrating local autoreactive plasma cell differentiation in joint biopsies captured from untreated early RA. Hence, plasma cell niches are not a consequence of chronic inflammation, but are already present at the time of diagnosis.

## Introduction

Rheumatoid arthritis (RA) is a chronic autoimmune disease that is characterized by inflammation of the peripheral joints^1^. Two thirds of the patients can be further distinguished by occurrence of autoantibodies in circulation^2^. These autoantibodies include Rheumatoid Factor, i.e. an anti-Fc gamma of IgM or IgA isotype, and anti-citrullinated protein antibodies (ACPA) that are clinically assessed by an anti-cyclic citrullinated peptide (anti-CCP) IgG ELISA. The autoantibodies often co-exist and can precede the clinical onset of RA by years^3–5^.

The synovial tissue of RA patients displays varying degrees of inflammatory infiltrates, which can be broadly subcategorized into three histological pathotypes: scarcely infiltrated by immune cells, diffusely infiltrated by immune cells or harboring ectopic lymphoid structures^6^. These lymphoid structures comprise T and B cells which can contribute to HLA class II-dependent immune responses that are associated with ACPA + disease^7^. While such immunological features in the inflamed synovia are well recognized in long-standing ACPA + RA, it is unknown whether in situ B cell differentiation into autoantibody producing plasma cells is a feature of longstanding inflammation or if it occurs already at the onset of RA.

The systemic autoimmunity of so called ‘at risk’ individuals with a positive serology test is not reflected in the joints, as such individuals lack signs of synovitis^8^. The healthy joint is devoid of lymphocytes and this has also been reported to be the case for autoantibody positive risk subjects^8^, supporting the notion that local plasma cells may not be an early phenotype in RA. Here, we studied joint biopsies from untreated early ACPA+ and ACPA− RA patients using a cutting edge integrated single cell and spatial transcriptomic approach, coupled with bioinformatics analysis. The affected synovial tissues harbored distinctive plasma cells and plasma cell survival niches already at the time of RA diagnosis. Moreover, we found shared clonalities between the synovial plasma cell and the patient-matched memory B cell compartments indicating local B cell differentiation. One CCP-positive monoclonal antibody was identified from the expressed plasma cell derived immunoglobulin sequences supporting the notion of disease-specific local B cell differentiation in the joints already at the time of diagnosis.

## Patients and methods

### Experimental design

Synovial tissue from Rheumatoid Arthritis (RA) patients were captured during 2018. The inclusion criteria were that biopsies were taken at the time of diagnosis and that patients were naïve to anti-rheumatic treatment. Since RA starts with manifestations in small joints, we initially sampled small joints such as the metacarpophalangeal joint. However, due to low B cell recovery from this site, we instead focused on sampling larger joints such as wrists and knees.

### Patients

All RA patients were recruited from the Rheumatology Clinic of the Karolinska University Hospital. The study was approved by the Swedish Ethical Review Authority. All biopsies were performed in accordance with the Helsinki Declaration and written informed consent was given by each patient before entering the study. Patients were instructed to withhold any NSAID treatment for five days before their clinical visit, at which diagnosis was made. Biopsies were captured from eight RA patients 1–2 days after their diagnosis. Clinical features of the patients are presented in Table 1. We selected three patients with high quality scRNAseq data and one additional patient for the spatial transcriptomics.

**Table 1:** Patient characteristics.

	All (n = 8)	ACPA+	ACPA+	ACPA−	ACPA−
Subjects (N/gender)	7 F/1 M	F	F	F	F
Age (median/range)	70 (23–80)	70–75	45–50	70–75	60–65
CCP-titer (range/cut-off 3)	4 + (80–300) 4 − (< 0.5–0.5)	300	> 300	< 0.5	0.5
RF status (+/−)	4+/4−	+	+	−	−
Tender joints, 28-count (median, range)	11 (8–16)	10	8	16	8
Swollen joints, 28-count (median, range)	10 (1–18)	16	7	14	11
Pain, global VAS (median, range)	75 (26–87)	87	50	84	83
CRP, mg/l (median, range)	14 (1–135)	81	4	14	135
ESR, mm (median, range)	49 (20–71)	44	36	56	71
DAS28-CRP (median, range)	5.4 (3.85–6.66)	6.66	4.56	6.4	6.4
DAS28-SR (median, range)	6.47 (5.07–7.28)	6.76	5.53	7.28	6.66
Time of biopsy (days after diagnosis)	2 (1–2)	2	2	2	2
Time of symptom (months before diagnosis)	6 (2–24)	24*	5.5	4	5

*This patient had knee pain 24 months prior to fulfilling an RA diagnosis that was ascribed as early knee arthrosis based on X-ray (no any alterations in small joints were found at this time point). She subsequently developed small joint arthritis 1 month prior to RA diagnosis.

### Sample processing

Arthroscopic biopsies were taken from large joints and ultra-sound guided biopsies from small joints of RA patients within two days of diagnosis.

One set of the biopsies was collected for scRNAseq in cold RPMI medium (Gibco) supplemented with 1% penicillin streptomycin and 1% glutamine. The tissue was digested in 4 mg/ml Collagenase A (Roche) and 0.1 mg/ml DNAse (Roche) in RPMI medium at 37 °C while shaking. After 1 h, ice cold RPMI medium supplemented with 10% FBS was added to stop the enzymatic reaction. The tissue was strained through a 40 µm mesh. Cells were washed and counted in ice cold PBS (Gibco). Cells were resuspended in 50 µl PBS and stained in antibody staining mixture containing 3 µl anti-CD19 BV421 (HIB19), 10 µl anti-CD3 FITC (UCHT1), 10 µl anti-CD14 FITC (M5E2), 5 µl anti-IgD BV605 (IA6-2), 5 µl anti-CD138 APC (MI15), 5 µl anti-CD27 PE-Cy7 (M-T271, all Becton Dickinson) and 12 µl 1% human serum for 30 min at 4 °C. Cells were washed, resuspended in 50 µl AnnexinV binding buffer and stained with 4 µl AnnexinV FITC for 10 min at RT. Cells were diluted in AnnexinV binding buffer. Cells were sorted on a Becton Dickinson influx cell sorter. The gating strategy is depicted in Supplementary Fig. 1. 3468 single cells were sorted into 384-well plates containing lysis buffer, before snap frozen. We submitted plates with ≥ 382 cells/donor for scRNAseq reducing our patient pool to five.

Another set of tissue pieces were snap frozen in isopentane prechilled with liquid nitrogen and stored at − 70 °C for immunohistochemistry, spatial RIN and 10X Visium analyses.

### Single cell library preparation, sequencing and analysis

Single cell libraries were prepared according to the SmartSeq2 protocol^9^. In brief, mRNA from single cells and ERCC spike-in were reverse transcribed using an Oligo(dT) primer, before a locked nucleic acid strand switch primer was added. cDNA amplification was primed by ISPCR primers and yields were assessed on an Agilent Bioanalyzer. The PCR product was tagmented using a Tn5 transposase integrating Read1 and Read2 sequences at the termini of the target DNA. Libraries were indexed using the Nextera XT DNA indices and assessed for concentration on the Agilent Bioanalyzer. Libraries were pooled per plate and sequenced on an Illumina NovaSeq with 2 × 100 bp paired-end reads yielding a median of 2.5 M reads/well. Each plate had two empty control wells.

FCS files were analyzed using FlowJo and R. Single cell data was extracted using InfluxIndex2CSV.

Raw reads were processed using FastQC. Adapter sequences were trimmed using TrimGalore and mapped using STAR. Low quality cells that resembled empty control wells in terms of quality metrics were excluded by assessing a threshold along the first principal component vector in R. The quality metrics included the Q30 score, the number of raw read sequences, the number of failed FastQC metrics, before and after trimming, the number and length of mapped reads, mapped genes and ERCC reads.

The ERCC spike-ins were labelled by the scran::isSpike function. We computed technical and biological size factors by using the scran::computeSpikeFactors and scran::computeSumFactors functions. We normalized the raw read counts of endogenous genes with the biological size factors, and that of spike-ins with the technical size factors using the scater::normalize function. We fitted a curve to the mean–variance values of spike-ins using the scran::trendVar function. The fitted value is used to estimate the technical component of the total variance of an endogenous gene. We used the scran::decomposeVar function to find genes with significantly higher variation than the fitted technical variation (biological variance > 0.5 and FDR ≤ 0.05). These 2747 highly variable genes were kept for UMAP dimensionality reduction using the scater::runUMAP function. Three clusters were visually identified. For cluster labeling, we used a k-means clustering identifying 5 clusters. One cluster was composed of naïve B cells, one cluster was composed of plasma cells and three clusters were joined and composed of memory cells. For differential gene expression analysis, we selected the top-20 differentially expressed genes by assessing the average log2FC of expression for each cluster against the other two.

### Immunohistochemistry

Cryostat sections (7 µm) from the four samples submitted to spatial transcriptomics analyses were fixed with 2% formaldehyde (Sigma) for 20 min. Sections were incubated with mouse anti-CD19 (HD37) mixed with mouse anti-CD20 (L26) antibodies from Dako Denmark A/S for validation of B cells according to a described protocol^10^. Isotype matched irrelevant antibodies were used as a negative control. The staining sections were examined using a Polyvar II microscope (Reichert-Jung, Vienna, Austria) and Leica Qwin IM500 software (Leica, Cambridge, UK).

### Spatial RIN analysis

The Spatial RIN assay was used to assess the quality distribution of RNA over the biopsies. The protocol was previously described^11^ (Supplementary Fig. 1). The sectioning thickness was set to 7 µm. Fixation was performed in 100% methanol in freezing conditions for 30 min and after staining and imaging, an 8 min permeabilization followed. The tissue removal step was performed for 1 h in 56 °C with interval shake using 4 × β-mercaptoethanol diluted in Buffer RLT Plus (QIAGEN). Further steps were performed as described.

### 10X Visium processing

The Visium Spatial Tissue Optimization Slide & Reagent kit (10X Genomics) was used to optimize permeabilization conditions for the fresh frozen tissue biopsies. The manufacturer’s protocol was followed and the optimal permeabilization time was determined to be 15 min.

A total of ten sections divided into four patient samples were sectioned with 7 µm thickness and mounted onto Visium Spatial Gene Expression Slides & Reagent kits (10X Genomics). Sequencing libraries were prepared following the manufacturer’s protocol, with permeabilization performed for 15 min, as established in the TO assay. The 10X Visium spatial transcriptomics libraries were prepared and sequenced on an Illumina NextSeq with the 150 cycles high output kit. The libraries were sequenced with 28 bp from Read1 and 120 bp from Read2, and at a depth of about 100 M reads/sample.

### 10X Visium data processing

Following demultiplexing of BCL files, the Read2 FASTQ files were trimmed using Cutadapt^12^ to remove full-length or truncated template switch oligo (TSO) sequences (AAGCAGTGGTATCAACGCAGAGTACATGGG) from the 5′ end and polyA homopolymers from the 3′ end. We required partial matches up to 5 bp or intact TSO sequences with a maximum of 3 errors. For the 30 homopolymer trimming, a sequence of 10 A’s was used regardless of the position in the read. The trimmed data were processed with the Space Ranger pipeline (version 1.0.0, 10X Genomics) and mapped to the GRCh38 v93 genome assembly.

### 10X Visium analysis

The spatial gene expression data was analyzed with the STUtility package^13^ and the Seurat package v.3.2.2^14,15^. Each section was individually normalized with the SCTransform function which uses a regularized negative binomial regression to transform the UMI count data. Thereafter donor integration was performed with the Harmony algorithm followed by a Shared Nearest Neighbor (SNN) construct graph and clustering using default settings. The clusters were colored and the Harmony embedded data was visualized in an UMAP and on the tissue sections. The raw counts of the clustered data underwent a differential gene expression analysis with logFC > 1 and adjusted p-value < 0.05 cut-offs to define sets of upregulated genes.

The factor analysis was run with a Non-negative Matrix Factorization specifying 20 factors on all batch corrected SCTransformed sections. The pathway analysis was performed with the BioPlanet 2019 tool^16^ on the top-20 genes from the factor analysis.

### Single cell and spatial data integration

To find significantly enriched genes for memory B cells and plasma cells from our data, and SC-B1, SC-B2 and SC-B4 from the Zhang et al. study^17^, we used Kruskal–Wallis Test with Dunn correction and performed p-value adjustment for multiple testing according to the Bonferroni method. As comparators, we only used the previously mentioned clusters as well as the cluster of naïve B cells from our data. Significantly enriched genes were selected based on average expression FC > 1 and adjusted p-value < 0.01, whilst the comparators were ignored. To find overlapping gene signatures of the clusters from both datasets, we used Fisher’s exact test under the H_0_ hypothesis that there are random associations between the significantly enriched B cell markers in both data sets. The single cell data from Zhang et al.^17^ was predicted on the spatial tissue data with the FindTransferAnchors and TransferData functions from the Seurat package with default settings. This was performed with SCTransformed data on both the single cell data set and the spatial data set. Spearman correlation was used as an estimate for co-localization of the SC-B2 and SC-B4 cells against all other single cell types. Besides, we showed raw counts for CXCL12 and VIM expression, respectively.

### B cell receptor reconstruction and expression

For B cell receptor reconstruction from the scRNAseq data, we used BraCeR^18^ assigning the sequences to the IMGT (Immunogenetics) database. The output tables were filtered based on completeness and expression levels of the paired V(D)J transcripts using a customized R script. Combined heavy and light chain sequences were aligned using Clustal Omega. The alignment was visualized using FigTree. Selected immunoglobulin heavy and light chain pairs were cloned into expression vectors^19^. Recombinant monoclonal antibodies were expressed in the Expi293 system (Thermo Fisher Scientific). Purified antibody concentration was determined by ELISA.

### Reactivity testing

Monoclonal antibody binding was investigated at 5 µg/ml. Anti-CCP2 ELISA (Euro Diagnostica) was performed according to manufacturer’s instructions. The serum (1:50 dilution) cut-off for positivity was 25units/ml. For the polyreactivity ELISA, soluble membrane protein (SMP) extract was coated at 5 µg/ml^19^. The ACPA was further characterized by a modified Vimentin peptide ELISA (Orgentec Diagnostika) containing citrulline, acetyl-lysine, homocitrulline, acetyl-ornithine or ornithine residues. The ACPA fine specificity was further evaluated using a custom-made peptide array (Thermo Fisher Scientific, ImmunoDiagnostics) containing citrullinated peptides and control antigens^20^.

### Statistical analysis

Details can be found in the respective methods section. Sample numbers are provided in figure legends. Differentially enriched genes were assessed using Kruskal–Wallis Test with Dunn correction. p-values were adjusted for multiple testing according to the Bonferroni method. Overlapping gene signatures were tested using Fisher’s exact test. Colocalization was assessed using Spearman’s R.

## Results

### Phenotype and tissue context of synovial B cells at the onset of RA

**Figure 1:**
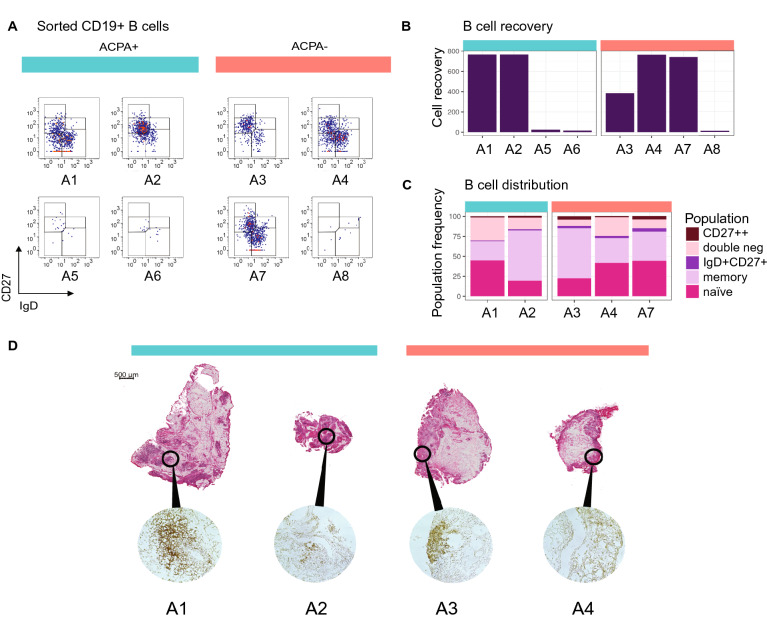
Number, phenotype and spatial distribution of synovial B cells in Rheumatoid Arthritis. (**A**) The flow cytometry panels show all CD3-CD14-AnnexinV-CD19+ single B cells from synovial biopsies of 4 ACPA+ and 4 ACPA− RA patients at the time of diagnosis in pseudocolor plots, where red color indicates data point overlap. A5 was taken from a metacarpophalangeal (MCP) 4 joint, A6 and A7 were taken from wrists. Remaining synovial biopsies are arthroscopic biopsies from knees. (**B**) 5 out of 8 biopsies exceeded a minimum of 382 B cells suitable for phenotypic characterization and scRNAseq analysis. (**C**) Memory B cells made up the largest population of synovial B cells (24–63%), while naïve cells were a bit fewer (20–45%). Double negative B cells had a variable range (8–29%), while a minority was CD27++ (1–4%) or of the unswitched IgD+ CD27+ memory phenotype (2–4%). (**D)** H&E-staining shows the tissue architecture of A1–A4. Immunohistochemistry staining with anti-CD19 and anti-CD20 antibody cocktail confirms the presence of B cells. ACPA+ samples are indicated by turquoise color, and ACPA− in orange.

Eight synovial tissue biopsies from small (MCP, wrists) and large (knee) joints from four ACPA+ and four ACPA− RA patients collected within two days of diagnosis were included in the study. A summary of the clinical features of the patients is displayed in Table 1. Following flow cytometric sorting for CD3-CD14-AnnexinV-CD19 + cells, we retrieved viable B cells from all samples (Fig. 1A) but the large joints yielded substantially more that the small joints, reflecting the amount of tissues obtained (Fig. 1B). The B cells from the cell rich tissues where phenotyped based on their IgD and CD27 protein expression. Hereby, 20–45% of the B cells displayed a naïve IgD+ CD27− phenotype, while 24–63% had a IgD−CD27 + classical memory phenotype, 1–4% were CD27++, 2–4% had an IgD+ CD27+ unswitched memory phenotype and 8–29% had an IgD− CD27− phenotype (Fig. 1C). These subsets were apparent irrespective of ACPA status of patients.

Cryopreserved biopsy samples were available from four subjects (A1–A4) for parallel studies focusing on the inflammatory architecture. Hereby, H&E-staining and immunohistochemistry (Fig. 1D) confirmed the presence of B cells in all samples with B cell-rich infiltrates especially around the vessels in samples A1 and A3, while samples A2 and A4 displayed fewer dispersed B cells.

### Single cell and spatial transcriptome analyses of synovial B cells

**Figure 2:**
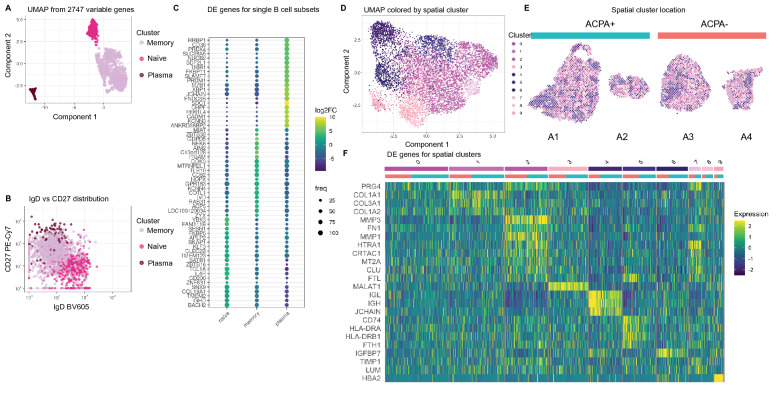
Identification of synovial B cell clusters and assessment of context relevant markers. (**A**) The UMAP dimensionality reduced B cell transcriptome from A2–A4 reveals the presence of a naïve, a memory and a plasma cell cluster. (**B**) The scRNAseq annotation translates back onto the expected phenotypic protein expression by flow cytometry. (**C**) Average log2FC and robustness of expression for differentially expressed (DE) genes of B cell transcriptomes show marker genes for naïve, memory and plasma cells. (**D**) UMAP on spatial transcriptomics data colored with the ten nearest neighbor clusters. (**E**) Clusters are shown on the tissue sections. ACPA+ samples are indicated by turquoise color, and ACPA− in orange. (**F**) Differentially expressed genes between the ten clusters (indicated by same colors as in (**D**). Cluster 4 shows genes strongly associated to plasma cells.

Altogether, we index-sorted 3468 individual B cells (14–764 cells/donor) from synovial biopsies from eight RA patients using flow cytometry (Supplementary Fig. 2). Five out of the eight samples had sufficient B cell numbers (> 382 cells) and were further processed for scRNAseq. Three out of the five passed QC; these originated from one ACPA+ and two ACPA− RA patients. In uniform manifold approximation and projection (UMAP) embedding, the B cells from patients A2–A4 distributed into three clusters. We identified 113 plasma cells (6%), 395 naïve B cells (21%) and 1388 memory B cells (73%, Fig. 2A). Notably, the plasma cell cluster was the most distinct, while the naïve and memory cell clusters were more similar. The plasma and memory B cell clusters had contributions from all three patients, while the naïve B cell cluster was mainly (95%) composed of cells from one of the ACPA− RA samples (A4). This is consistent with the large naïve B cell population (42%) observed for this patient in the flow cytometry data. The memory cell cluster was influenced by patient specificity (Supplementary Fig. 3). Assignment of the cluster labels from the scRNAseq back into the flow cytometry distribution of IgD vs CD27 recapitulated the index labels from the sort validating our approach (Fig. 2B). Reassuringly, we found that the naïve cell cluster fell into the IgD+ CD27− region, while the memory cluster was IgD− CD27+ and as expected, plasma cells were traced back as IgD− CD27++. We did not find enrichment of *ITGAX* (encoding CD11c) in the double negative population, which would indicate autoimmunity-associated B cells (Supplementary Fig. 4). Instead, the cells defined by the double negative gate in the flow cytometric analysis mapped to all clusters in the single cell transcriptomic data (Supplementary Fig. 5). Figure 2C depicts the top-20 most differentially expressed genes (DEGs) between the three clusters. The most distinct features expressed by the plasma cell population included the plasma cell markers *XBP1*, *SDC1* (encoding CD138), *SLAMF7* and *PRDM1* (encoding BLIMP-1). Additionally, this cluster had increased expression of *JCHAIN*, an indication of high antibody protein production. In contrast, the memory B cell cluster was characterized by *ITGAM* encoding CD11b and *GPR138* encoding EBI2, which is typical of memory B cells. The naïve B cell cluster was characterized by *IGHD* and *IL4R* expression encoding IgD and IL4 receptor which are representative of naïve resting B cells.

Two ACPA− and two ACPA+ tissues were processed also for spatial transcriptomics. Hereby, we generated comprehensive mRNA data on 18,199 genes over 7334 spots distributed on consecutive cryosections. The number of transcripts per spot was heterogeneous as expected from the tissue morphology observed in the H&E-staining. We verified that RNA quality was adequate also in less cell dense areas by assessing spatial RIN scores (Supplementary Fig. 1). After data integration and subjection to UMAP, we identified 10 clusters (Fig. 2D) that spatially mapped into the sections (Fig. 2E). These clusters matched the different morphological areas (Fig. 1D). The dark blue clusters (4, 5 and 6) overlapped with the cell dense lymphocytic areas and were present in all four patients. The heatmap (Fig. 2F) shows the top DEGs of the 10 clusters, to which both the ACPA+ and ACPA− samples contributed. We additionally subjected the data to a PCA visualizing that the patient subgroups were largely overlapping (Supplementary Fig. 6). Cluster 4, which was in proximity of the infiltrate regions and the B cell-rich areas had a strong expression of *JCHAIN*, IGH, IGL, *MZB1*, *SDC1* and *XBP1*, suggesting the presence of plasma cells in these locations (Supplementary Table 1). Cluster 5 had a strong signal for immune cells with high expression of HLA class II genes and *CD74* indicating antigen presentation. Close by cluster 4 and 5 was cluster 6 with a weaker immune signature although containing genes encoding the chemokine CCL19 known to attract B and T cells.

The remaining clusters overlapped with areas outside the infiltrates, and contained genes associated to connective tissue such as collagen, metallopeptidases, fibronectin and proteoglycan as well as inflammatory features such as complement factor C3. This analysis revealed the heterogeneity of the tissue even in seemingly homogenous morphological areas (Supplementary Table 1).

### B–T cell interaction in the RA joint tissue

**Figure 3:**
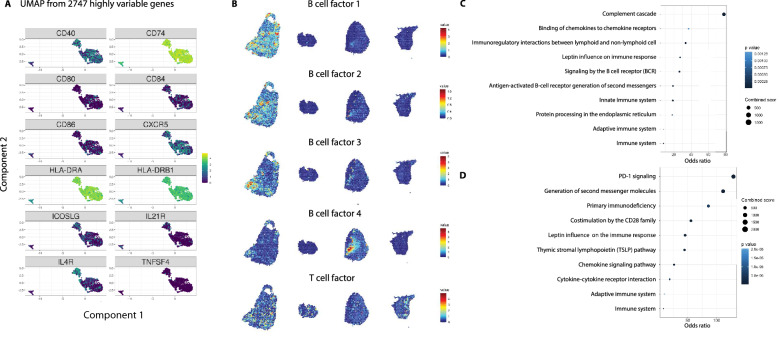
T cell interaction as a means of B cell maturation in the synovial tissue. (**A**) Expression of genes associated with antigen presentation and costimulation are displayed on the three major clusters formed from the single B cell RNAseq. (**B**) Upon factor analyses, four B cell factors and one T-B cell interaction factor are shown with factor activities. These signatures overlap in distinct morphological areas of both ACPA− and ACPA+ tissue sections. (**C**) Pathway analysis from the top-20 genes for B cell and (**D**) T-B cell interaction factors (T cell factor) in B showed BCR signaling and PD-1 signaling, respectively.

All B cell subsets expressed high amounts of *CD74* encoding the HLA invariant chain as well as the *HLA-DRB1* and the paired *HLA-DRA* chain genes implicating antigen-presentation capacity (Fig. 3A, Supplementary Fig. 7). Based on this observation, we further investigated other B cell transcript expressions indicating T cell interaction. We found expression of the genes encoding the costimulatory molecules CD40, CD80, CD86, CD84 and also low expression of *TNFSF4* encoding CD134 (also known as OX40L). Furthermore, primarily naïve B cells expressed *ICOSLG*, *IL4R* and *IL21R*. IL21 receptor signaling is important for B cell proliferation and class switch recombination as well as for plasma cell differentiation. Robust expression was also found for *CXCR5*, which encodes the receptor for *CXCL13* produced by peripheral helper T cells.

From the tissue transcriptional data, factor analysis was performed, and 20 different factors and their driver genes could be identified (Supplementary Fig. 8). Factorization is used to represent the data in lower dimensional space. The factor activity describes the relative contribution of each spot to the factor. Five factors showing a B cell and T-B cell interaction signature (6, 5, 8, 13, 17) are presented in Fig. 3B. The associated genes underwent pathway analysis through NCATS BioPlanet 2019 (Fig. 3C,D). This analysis revealed BCR signaling for the factors denoting increased B cell activity and PD-1 signaling as the top hit in the T–B cell interaction pathways. Again, both the ACPA+ and ACPA− biopsies displayed clear signals of B-T cell crosstalk.

### Transcriptomic profile and spatial context of memory B and plasma cells

**Figure 4:**
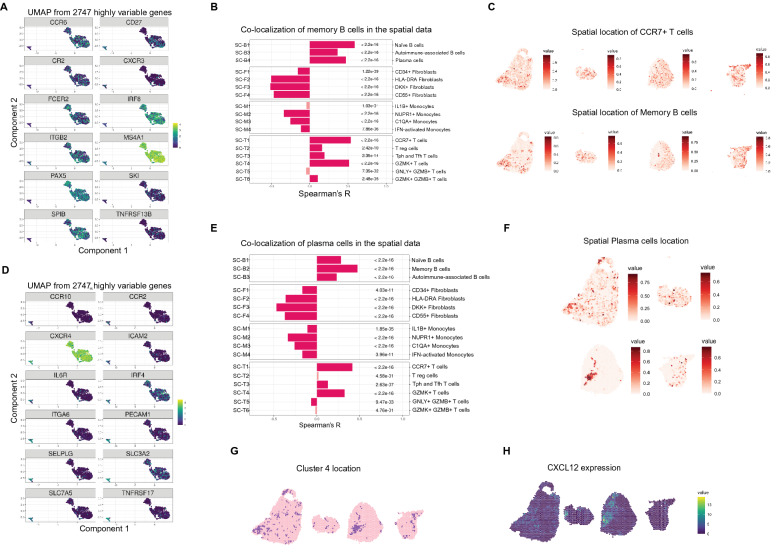
Memory B and plasma cell niches in synovial tissue at the time of diagnosis. (**A**) The memory cell markers are either shared with the naïve cells, such as *CR2* encoding CD21 or *FCER2* encoding CD23, or they are shared with the plasma cells, such TACI encoded by *TNFRSF13B*. (**B**) We determined correlation between the predicted location score of memory B cells (SC-B2) and the other clusters in the spatial data^17^. (**C**) The spatial distribution of memory B cells (SC-B2) and *CCR7* + T cells (SC-T1) show a dispersed but correlating pattern. (**D**) We find expression of genes in plasma cells that are important for matrix interaction such as *ICAM2* and *ITGA6* encoding CD49f. We also find expression of *CXCR4* and of BCMA encoded by *TNFRSF17,* which are important for the plasma cell niches. (**E**) We determined correlation between the predicted location score of plasma cells (SC-B4) and the other clusters in the spatial data^17^. (**F**) Plasma cells are confined and adjacent to the lymphocyte infiltrates. (**G**) We find overlap between the designated and the predicted plasma cell cluster. (**H**) Additionally, we find high CXCL12 expression in the plasma cell areas suggesting a niche in the inflamed tissue.

All memory B cell-associated markers were either shared with naïve B cells or with plasma cells (Fig. 4A, Supplementary Fig. 9). *CCR6*, *CR2* (encoding CD21), *FCER2* (encoding CD23), *ITGB2* (encoding CD18) and elevated expression of *MS4A1* (encoding CD20) were shared with the naïve B cell cluster, while *TNFRSF13B* (encoding TACI), *CD27* and *CXCR3* expression was shared with the plasma cell cluster. Also, gene expression of the transcription factors IRF8, PAX5 and Spi-B as well as the transcription co-regulator SKI were shared with the naïve B cell cluster.

Next, we investigated the spatial context of memory B cells. In order to enrich our findings, we used previously published single cell signatures by Zhang et al.^17^. Similarity of our and their memory B cell (SC-B2) signature was validated by Fisher’s exact test under the H_0_ hypothesis that there are random associations between the significantly enriched memory B cell markers in both data sets (OR = 99.6, p-value < 2.2e−16, Supplementary Table 2). We used Spearman’s R to assess co-localization of memory B cells (SC-B2) with the other described single cell subtypes including other B cell, fibroblast, monocyte and T cell subtypes in the spatial data (Fig. 4B,C, Supplementary Fig. 10). Memory B cells (SC-B2) mostly co-occurred with other B cells (0.37 ≤ R ≤ 0.59) implicating an increased likelihood for clonal expansions, and with T cells (0.11 ≤ R ≤ 0.54, Fig. 4B) implicating an increased likelihood for T cell interaction. Notably, the memory B cells (SC-B2) co-localized with the T peripheral or follicular helper cell subset (SC-T3, R = 0.20), but also with *CCR7* + T cells (SC-T1, R = 0.54) and *GZMK* + T cells (SC-T4, R = 0.51). There was an inverse correlation for co-localization with most fibroblast (− 0.51 ≤ R ≤ − 0.16) and monocyte (− 0.34 ≤ R ≤ − 0.12) subsets.

We found terminally differentiated plasma cells in the rheumatoid synovium of both ACPA+ and ACPA− RA patients at the time of diagnosis. Besides the top-20 DEGs (Fig. 2C), plasma cells were further distinguished by expression of genes that are important for matrix interaction, such as *ICAM2* and *ITGA6* encoding CD49f (Fig. 4D, Supplementary Fig. 11). Additionally, plasma cells expressed *CCR2*, *CCR10* and *CXCR4*, which is important for the CXCL12-dependent plasma cell survival niche. Still, *CXCR4* expression was similarly to *IRF4* expression not exclusive to plasma cells. A gene that was more exclusive to plasma cells and also mediates plasma cell survival is *TNFRSF17* encoding the APRIL receptor BCMA. Additional markers that associated with plasma cells include *IL6R*, *PECAM1* (encoding CD31), *SELPLG* (encoding CD162) and the large neutral amino acid transporter, CD98, formed by the gene products of *SLC3A2* and *SLC7A5*.

Similar to the analysis for memory B cells, we took advantage of the previously published scRNAseq signatures^17^ to describe the location and context of plasma cells in our spatial transcriptomic data. The Fisher’s exact test validated the overlap between the DEGs in our plasma cell cluster and the previously described plasma cell (SC-B4) cluster (OR = 45.1, p-value < 2.2e−16, Supplementary Table 3). As memory B cells (SC-B2) and plasma cells (SC-B4) were highly co-localized (R = 0.48), we observed a similar pattern for co-localization for plasma cells (SC-B4) as for memory B cells (SC-B2, Fig. 4E). Plasma cells (SC-B4) co-localized with other B cell subsets (0.23 ≤ R ≤ 0.48) and several subsets of T cells namely *CCR7* + T cells (SC-T1, R = 0.42), T peripheral or follicular helper cells (SC-T3, R = 0.13) and GZMK + T cells (SC-T4, R = 0.33). In contrast, the Spearman correlation was inverse for GNLY + GZMB + T cells (SC-T5, R = − 0.07), fibroblasts (− 0.47 ≤ R ≤ − 0.17) and monocytes (− 0.34 ≤ R ≤ − 0.11). Spatially, the plasma cell (SC-B4) cluster was more distinct than the memory B cell (SC-B2) cluster (Fig. 4C,F, Supplementary Fig. 12). The likelihood of finding plasma cells in the tissue was increased in the vicinity of lymphocyte infiltrates as also the correlation estimates were somewhat lower between plasma cells (SC-B4) and B or T cells compared to memory B cells (SC-B2) and B or T cells. Moreover, we observed a high overlap with the predicted location for plasma cells (SC-B4) and the spatial cluster 4 (Fig. 2F), which we concluded to have a plasma cell signature (Fig. 4G). Additionally, we found a high overlap with *CXCL12* expression in the tissue (Fig. 4H). The CXCL12-CXCR4 axis is indispensable for plasma cell maintenance^21^ and our results suggest that this axis is active already at the time of diagnosis.

### Expression, clonality and reactivity of plasma cells

**Figure 5:**
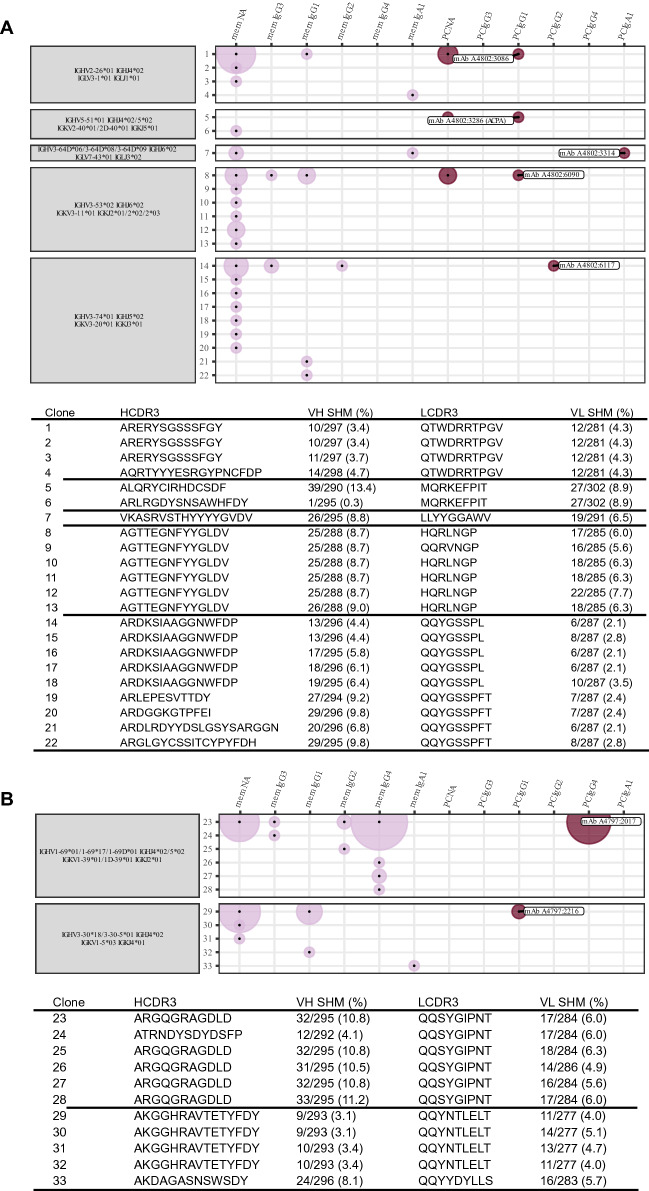
Clonotype characterization of synovial plasma cell derived antibodies. (**A**) We show clonotypes for five plasma cell derived monoclonal antibodies from A2. Each clonotype is defined by identical heavy and light chain V and J gene usage (grey boxes). Each clone with a unique sequence for the combination of heavy and light chain is assigned a number (y-axis). This sequence is described by the SHM and the complementary determining region 3 (CDR3) for the heavy and light chain in the table below. The x-axis and dot color depict the memory B (mem) or plasma cell (PC) cluster identity of the clone members by transcriptomic profile. It additionally defines the heavy chain isotype of the clone members in the order of possible class switch recombination from left to right. NA means not available from the data. The area of the dots is proportional to the number of identified members; e.g. the largest area within the first clone is equivalent to 16 members while the smallest area is equivalent to 1 member. (**B**) The plot shows the equivalent clonotype information that is shown in panel (A) for the two plasma cell derived monoclonal antibodies from A3.

Of the 113 identified plasma cells and their scRNAseq data, we reconstructed the full BCR sequence of all and further expressed antibodies corresponding to 9 unique clones, 3 clones with 2 cells each, 1 clone with 4 cells, 1 clone with 5 cells and 1 clone with 21 cells (Fig. 5A,B, Supplementary Fig. 13). 9 of these 15 clones were found to have sibling cells with an identical variable and constant chain pair within the memory B cell compartment. The identified clonotypes of biopsy A3 appeared to be less polyclonal than those of A2 and A4. For the largest clone, we ruled out potential index-hopping that might mis-assign BCR sequences to neighboring wells (Supplementary Fig. 14). The 5 clonotypes of biopsy A2 comprised 70 out of 753 (9%) memory and plasma cell members, the 2 clonotypes in A3 comprised 82 and 32 of 371 (22% and 9%) memory and plasma cell members and the 7 clonotypes of biopsy A4 comprised 38 out of 377 (10%) memory and plasma cell members, respectively. The expressed antibodies hence originated from all three biopsies with 5 antibodies derived from A2, 2 from A3, and 8 from A4.

**Figure 6:**
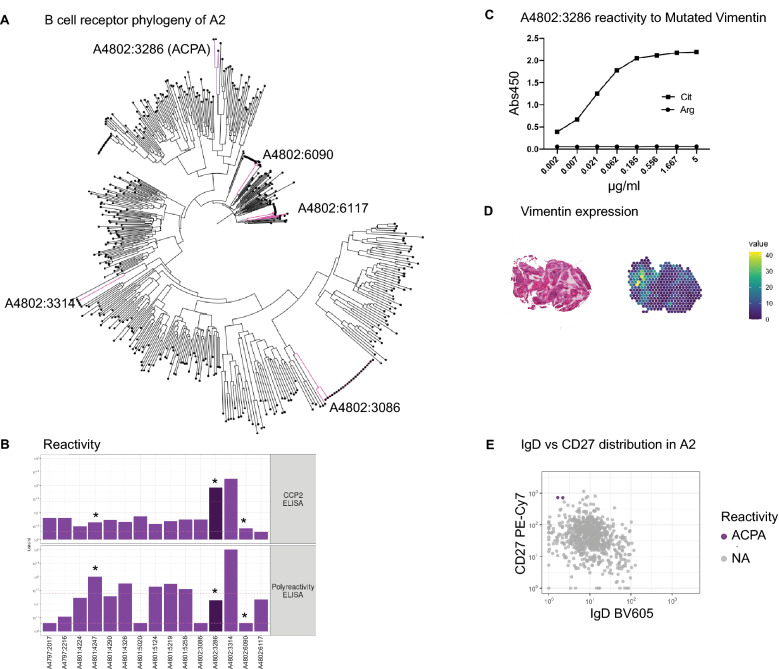
Reactivity landscape of synovial plasma cell derived antibodies. (**A**) Phylogenic tree of the B cell receptor clones of one of the ACPA positive individuals (A2). Clonotypes from figure are highlighted. (**B**) One of the monoclonal antibodies (dark purple) was identified as an anti-citrullinated protein antibody (ACPA) based on being CCP positive (upper panel), but not polyreactive (lower panel). Several antibodies showed polyreactivity. *Indicates insertion of N-linked glycosylation sites by SHM. (**C**) The A4802:3286 ACPA has strong reactivity towards mutated citrullinated vimentin peptide. (**D**) Vimentin is expressed within the corresponding tissue analyzed by spatial transcriptomics. (**E**) The A4802:3286 ACPA derives from two identical IgD− CD27++ plasma cells.

Figure 6A shows the 5 antibody clonotypes from the ACPA+ individual from whom the monoclonal ACPA was identified embedded in a phylogenetic tree with all recovered paired full length BCR sequences from that individual. All 5 clonotypes originated from expanded clones. Two of the recombinantly expressed monoclonal antibodies from the ACPA+ patient, A4802:3286 and A4802:3314, gave positive signals in the anti-CCP2 ELISA, while the other antibodies were nonreactive (Fig. 6B). However, A4802:3314 (an IgA1 and IgL3 clone) was further positive in the poly-reactivity ELISA and was therefore not defined as an ACPA. To continue investigating the reactivity of the A4802:3286 ACPA clone, we examined its potential multi-reactivity towards other post-translational modifications using the Orgentec clinical test containing a mutated vimentin backbone peptide. None of the other expressed mAbs showed reactivity in this test (data not shown). For the ACPA clone, the citrulline reactivity was positive in serial dilution down to a concentration of 2 ng/ml (Fig. 6C, Supplementary Fig. 15). In contrast, this clone was negative for other commonly studied citrulline specificities on a multi-peptide array (Supplementary Fig. 16). Vimentin is a classical RA candidate autoantigen, both as a citrullinated autoantigen and in a mutated citrullinated form, however our spatial transcriptomics data (of 120 bp reads) did not allow us to study the vimentin mutation in our biopsies. Still, we examined the level of gene expression and found abundant expression of vimentin on the patient specific section (Fig. 6D).

The A4802:3286 ACPA clone was of the IgK and IgG1 isotypes and originated from two identical CD27++ IgD− plasma cells in the original flow cytometry data (Fig. 6E). Furthermore, somatic hypermutation (SHM) had introduced two glycosylation sites in the antigen binding fragment (Fig. 6B, Supplementary Fig. 17), which is a common feature amongst ACPAs^22^. Out of the studied plasma cell clones, the A4802:3286 ACPA also had the highest level of SHM for both the heavy and light chains with 39 and 27 mutations, respectively (Fig. 5A). The ACPA clonotype used IGHV5-51*01, IGHJ4*02/5*02, IGKV2-40*01/2D-40*01 and IGKJ5*01 and had 3 members in total; one identical plasma cell sibling and one similar memory cell member with an identical light chain and a similar heavy chain. To summarize, we describe a monoclonal ACPA derived from two identical plasma cells from synovial tissue of early RA with a narrow reactivity pattern to a citrullinated RA antigen.

## Discussion

Here, we investigated the B cell compartment of joint biopsies from two ACPA+RF+ and two ACPA−RF− RA patients obtained within two days of diagnosis. We visualized the localization of B cell subsets within the biopsies and characterized them with regard to both T cell crosstalk and plasma cell survival niches. Moreover, based on the paired immunoglobulin sequences, we identified B cell clonotypes shared between the memory and plasma cell subsets. One of the expressed monoclonal antibodies from these joint-derived plasma cells demonstrated citrulline autoreactivity. Hence, all the characteristic features of RA known from established RA were evident in the joint already at the time of diagnosis.

In our unique approach, we integrated single cell resolution and spatial transcriptomic data from patients at the time of diagnosis, while previous studies^17,23,24^ analyzed single cells from dispersed synovial tissue from RA or osteoarthritis patients at mixed time points of the disease. We embedded parts of those studies in our analysis to enrich our findings with respect to the memory B and plasma cell context. In line with the study by Zhang et al.^17^ and Scheel et al.^23^, we also found that not all patients presented with high amounts of leukocytes or specifically B cells in the biopsy. In our experience, B cell yields correlated with disease activity at the time of sampling as well as with amount of tissue, but was independent of ACPA status.

We describe the presence of naïve, memory and plasma cells in the disease-affected synovial tissue of both ACPA+RF+ and ACPA−RF− RA patients. N.B. not all biopsies contained a robust B cell infiltration, but the ACPA status did not appear to be driving this difference. This is intriguing since it has been hypothesized that ACPA+ RA patients would have a stronger B–T cell drive in their disease based on the genetic HLA-DR association and the presence of autoantibodies. In contrast, RF marks the result of a different antibody response that is rather independent of the B-T cell drive^25^. Thus, we speculate that the RF response does not develop in the B cell rich synovial biopsies studied here. Our data demonstrated that the inflamed joint is an attractive site for B cells and plasma cell differentiation by B-T cell interaction, also in ACPA-RF- RA patients i.e. potentially also for disease-unspecific immune responses. Indeed, while ACPA+ RA patients respond better to B and T cell targeted therapies, ACPA− RA patients can also benefit from such therapeutic interventions^26,27^. It is of course possible that the B cell niche is less sustained in ACPA− patients following anti-inflammatory therapy since they would lack the link with locally expressed autoantigens.

Based on our transcriptomic data, we could identify many shared features between ACPA+ and ACPA− RA patients at this early time point before therapeutic intervention. Still, and importantly we only found citrulline autoimmunity within the ACPA + RA joint and the ACPA-producing plasma cell was found in two copies suggesting that it had expanded and differentiated locally. Hence, we found support for a local immunological B cell maturation process via T cell help involving PD-1 and CXCL13 as previously implicated for the joint of patients with longstanding ACPA+ RA by Rao et al.^28^. Another autoimmunity-related B cell feature which has received attention in recent years is atypical memory, most prominently in SLE^29^ but also in RA^30^. Although we found double negative B cells in the biopsies, they did not show the profile associated with age-associated B cells including CD11c and T-bet, supporting the notion that this B cell subset is instead associated with chronic immune responses or even immunosuppressive therapy, a stage that these RA subjects have not yet reached.

Additionally, we describe the nature of memory B cells and plasma cells in the joint and find supporting niche structures. Our data suggest BAFF and APRIL dependent survival of terminally differentiated B cells at the site of inflammation already at the time of RA diagnosis. Additionally, we find strong CXCR4 expression in memory B and plasma cells and elevated CXCL12 expression in the supporting tissue. Such a supporting niche has not been previously described at the site of joint inflammation, but is well known to be crucial for plasma cell survival in the bone marrow^31^. However, we could not identify co-localization signals between plasma cells and potentially supporting fibroblasts, which may be an effect of the compromised spatial resolution down to single cell level. In conclusion, we present evidence for immune maturation as well as maintenance of potentially pathogenic autoreactive memory and plasma cells in the rheumatic joint already at the onset of the disease.

While our approach to combine single cell and spatial transcriptomics for two ACPA− and two ACPA+ samples gives a detailed picture, the small sample size is limiting when drawing conclusions about the disease. We must acknowledge that the biopsies we have studied are not representative for the full spectrum of RA, which is a heterogeneous disease when it comes to the composition of the joint inflammation^6^. Additionally, one ACPA+RF+ and one ACPA-RF- patient included in our spatial transcriptomics study were non-responders to methotrexate, underscoring the difficulty in predicting the longterm outcome of this disease. Recent data suggest that many treatment refractory RA patients are found in patient with a so called pauci-immune histology^32^, i.e. lacking lymphoid and macrophage infiltrates. Our study did not include such samples and was instead biased to the lymphoid rich pathotype. Still, our study provides data from B cell rich synovitis that can be utilized for further studies, just as we made use of the Zhang et al. data^17^ in our analyses.

Intriguingly, we found a new plasma cell derived ACPA that displays some of the classic features previously attributed to RA autoantibodies such as N-linked glycosylation sites in the Fab fragment introduced by SHM^22,33,34^. However, this new tissue plasma cell-derived ACPA appears unique with a narrow pattern of reactivity compared to previously described ACPA^35–40^. The A4802:3286 ACPA clone reacted strongly to a mutated vimentin peptide originally identified in RA joint tissue^41^.

60% of our plasma cell derived monoclonal antibodies originated from expanded clones with representatives in both the memory and plasma cell compartment. Thus, while our data suggests a local B cell differentiation process and a local memory recall response, egression from the circulation may be an alternative explanation for the observation of clonal expansions in the joints.

Furthermore, we found six polyreactive plasma cell clones. Polyreactivity among mutated monoclonal antibodies has been reported in many different settings e.g. HIV infection and vaccination^42,43^. It has previously been demonstrated that there is a pool of polyreactive switched memory B cells that could originate from bypassing a tolerance checkpoint even in healthy donors^44^. We speculate that differentiation of these polyreactive, switched memory B cells into plasma cells perpetuates RA locally and systemically.

In summary, our study included synovial biopsies from untreated RA patients sampled within two days of diagnosis, hence the transcriptomic data we report are not biased by treatments such as steroids and disease-modifying anti-rheumatic drugs (DMARDs) that most RA patients receive. We find many similarities to other data sets analyzed much later in the disease process demonstrating that immune pathways implicated in disease pathogenesis are at play already at this early time point. The prominent lymphocyte infiltrations implicate local B cell-T cell interaction and the immunoglobulin sequence analyses demonstrate local plasma cell differentiation. This was true for both, ACPA+ and ACPA− individuals. Amongst the plasma cells in the inflamed joint, we identified a clonally expanded cell expressing an ACPA with strong reactivity to a modified citrullinated vimentin peptide, a specificity linked with osteoclast activation^39^. Future studies will show if this new ACPA also has functional capacities. Plasma cell survival niches in the joint were found present already at time of RA diagnosis supporting the notion that such structures are attractive therapeutic targets in RA^45^ or even individuals at risk of developing RA^46,47^.

## Supplementary Information

Supplementary Information.

## Data Availability

Raw sequencing data is available on the European Genome-Phenome Archive under the accession number EGAS00001005144; this dataset includes 3414 paired single cell sequencing fastq files derived from synovial B cells of 5 early Rheumatoid Arthritis patients. There are 764 paired fastq files for patient A1, 764 paired fastq files for patient A2, 382 paired fastq files for patient A3, 764 paired fastq files for patient A4 and 740 paired fastq files for patient A7. Additionally, there are paired fastq files for 18 empty control wells giving a total of 3432 paired-end read fastq files. Count matrices, cluster labels and BCR sequences are available through an MTA upon request from the authors.
