# Supplementary material for: Integrated single cell and spatial transcriptomics reveal autoreactive differentiated B cells in joints of early rheumatoid arthritis
Source: Sci Rep. 2022 Jul 13;12:11876. doi: 10.1038/s41598-022-15293-5 (PMC9279471; doi:10.1038/s41598-022-15293-5)
Supplement: Supplementary file 1 — Supplementary Information. [file 41598_2022_15293_MOESM1_ESM.pdf]

Supplementary Materials for Multimodal Synovial B Cell Sequencing in Arthritis

Full title: Integrated Single Cell and Spatial Transcriptomics Reveal Autoreactive Differentiated B Cells in Joints of Early Rheumatoid Arthritis

Authors: Uta Hardt[1,2,#,*], Konstantin Carlberg, PhD[3,#], Erik af Klint, MD, PhD[1], Peter Sahlström[1], Ludvig Larsson, PhD[3], Annika van Vollenhoven[1], Susana Hernandez Machado[1], Lena Israelsson[1], Khaled Amara, PhD[1], Karine Chemin, PhD[1], Marina Korotkova, MD, PhD[1], Gunilla B Karlsson Hedestam, PhD[2], Anca I Catrina, MD, PhD[1], Sarah A Teichmann, PhD[4], Patrik L Ståhl, PhD[3], Vivianne Malmström, PhD[1,*]

Author affiliations: [1]Division of Rheumatology, Department of Medicine Solna, Center for Molecular Medicine, Karolinska Institutet, Stockholm, Sweden and Karolinska University Hospital, Stockholm, Sweden. [2]Department of Microbiology, Tumor and Cell Biology, Karolinska Institutet, Stockholm, Sweden. [3]Department of Gene Technology, KTH Royal Institute of Technology, Science for Life Laboratory, Stockholm, Sweden. [4]Wellcome Sanger Institute, Wellcome Genome Campus, Hinxton, Cambridge CB10 1SA, United Kingdom. [#]These authors contributed equally.

Corresponding authors: Uta Hardt, PhD student, Departments of Medicine, Solna, and Microbiology, Tumor and Cell Biology, Karolinska Institutet, uta.hardt@ki.se, Tel: +46 8 524 869 43, Dr. Vivianne Malmström, Professor, Department of Medicine, Solna, Center for Molecular Medicine, Karolinska Institutet, vivianne.malmstrom@ki.se, Tel: +46 8 517 756 09

This file includes: Figs. S1 to S17**,** Tables S1 to S3

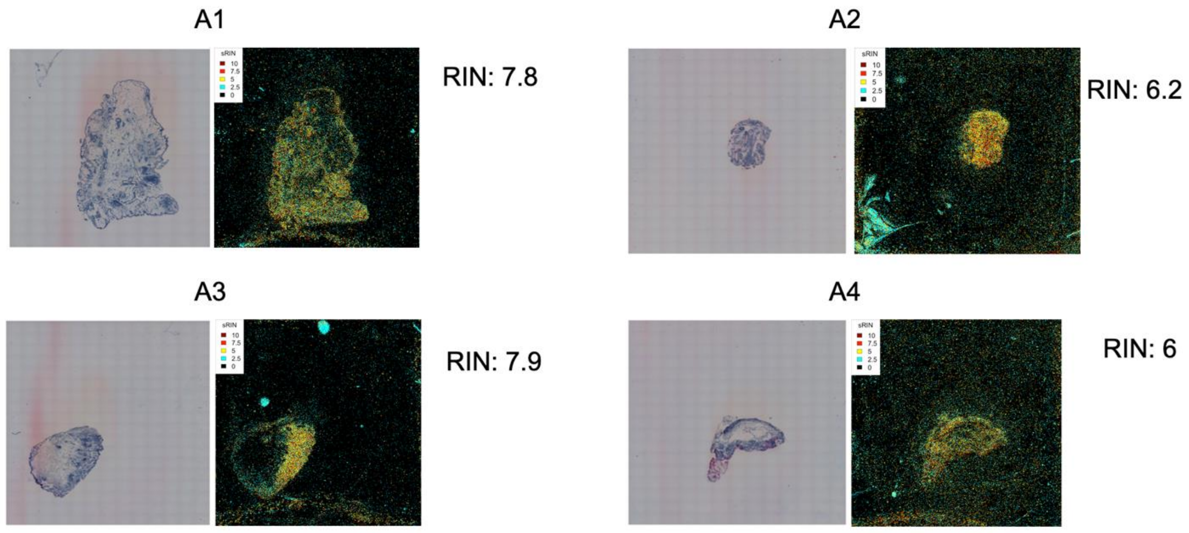

Fig. S1.

Spatial RIN. Heatmap over the spatial RIN scores with bulk RIN values from extracted material shown next to the heatmaps

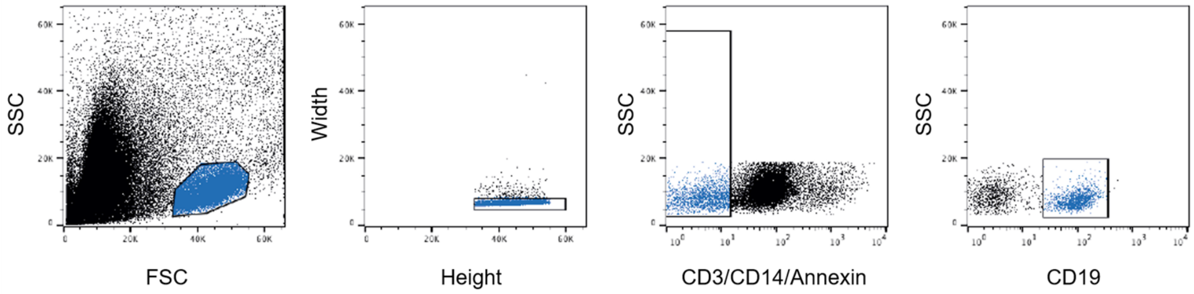

Fig. S2.

The gating strategy for the B cell sort is shown. We selected AnnexinV-CD3-CD14-CD19+ single lymphocytes.

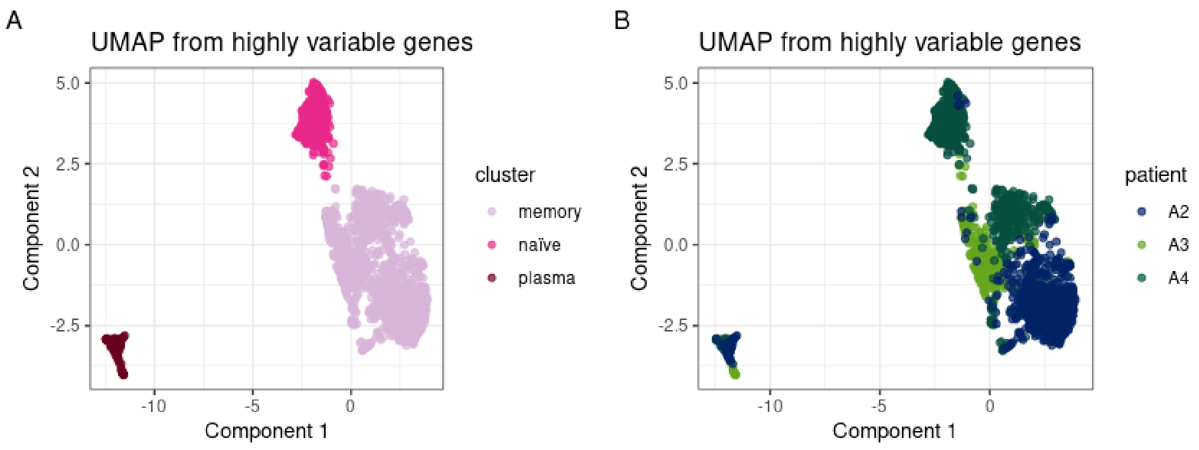

Fig. S3.

UMAP of scRNAseq data coloured by **(A)** cell cluster and **(B)** patient contribution.

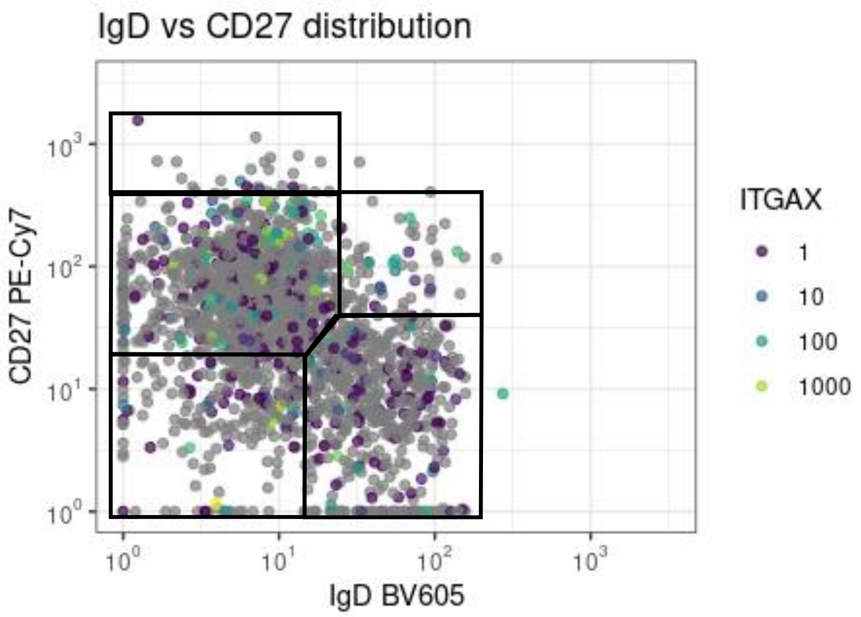

Fig. S4.

ITGAX (encoding CD11c) expression was projected onto the IgD vs CD27 distribution.

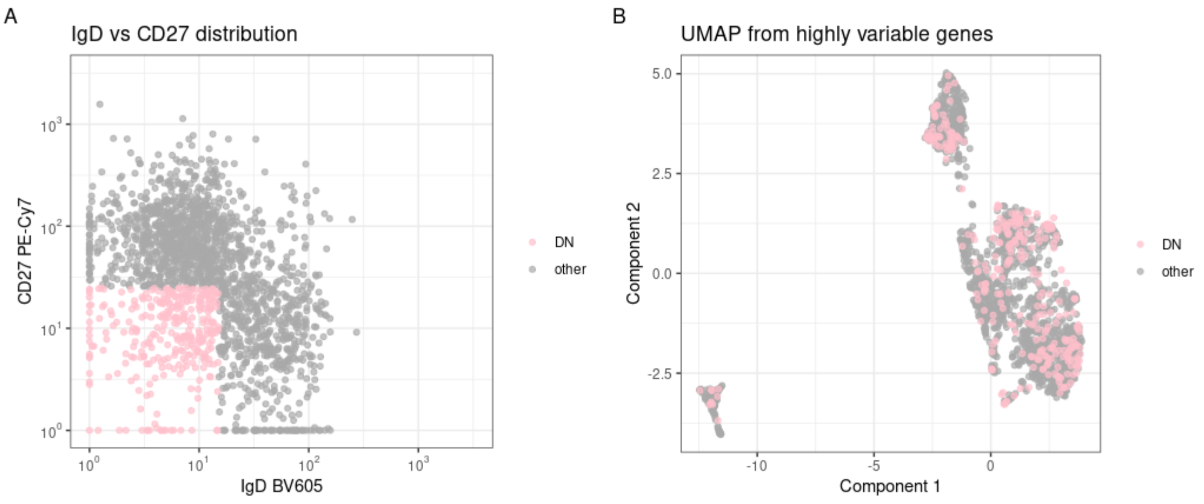

Fig. S5.

The double negative population in the scRNAseq data is shown.

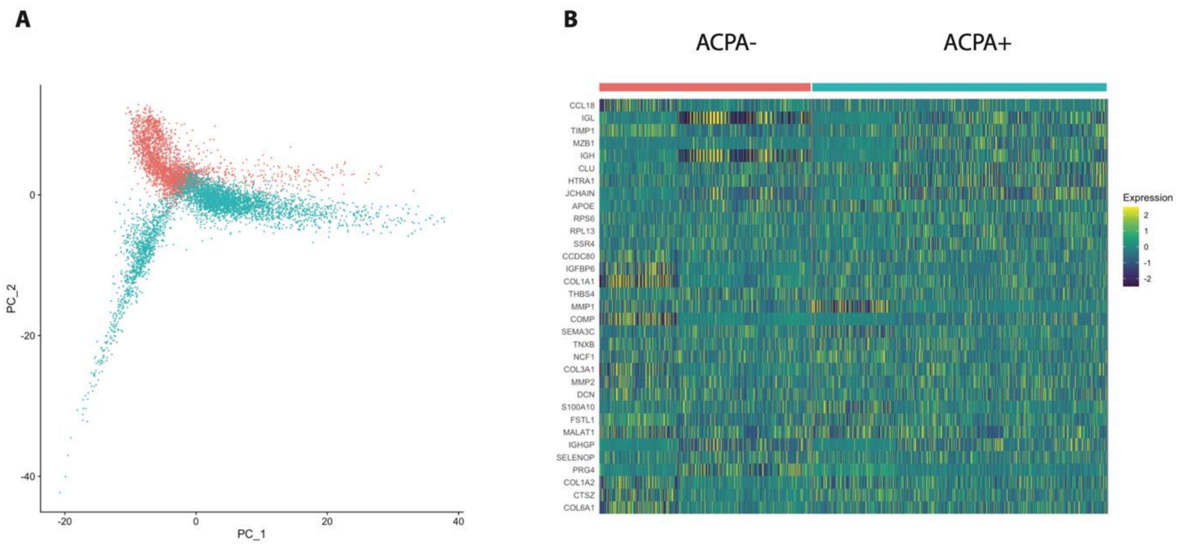

Fig. S6.

(A) PCA labelled by serotype (red ACPA-, turquoise ACPA+) showing overlaps and separation between data points for each group. **(B)** Heatmap over the marker genes for each group with little differences between ACPA+ and ACPA- RA.

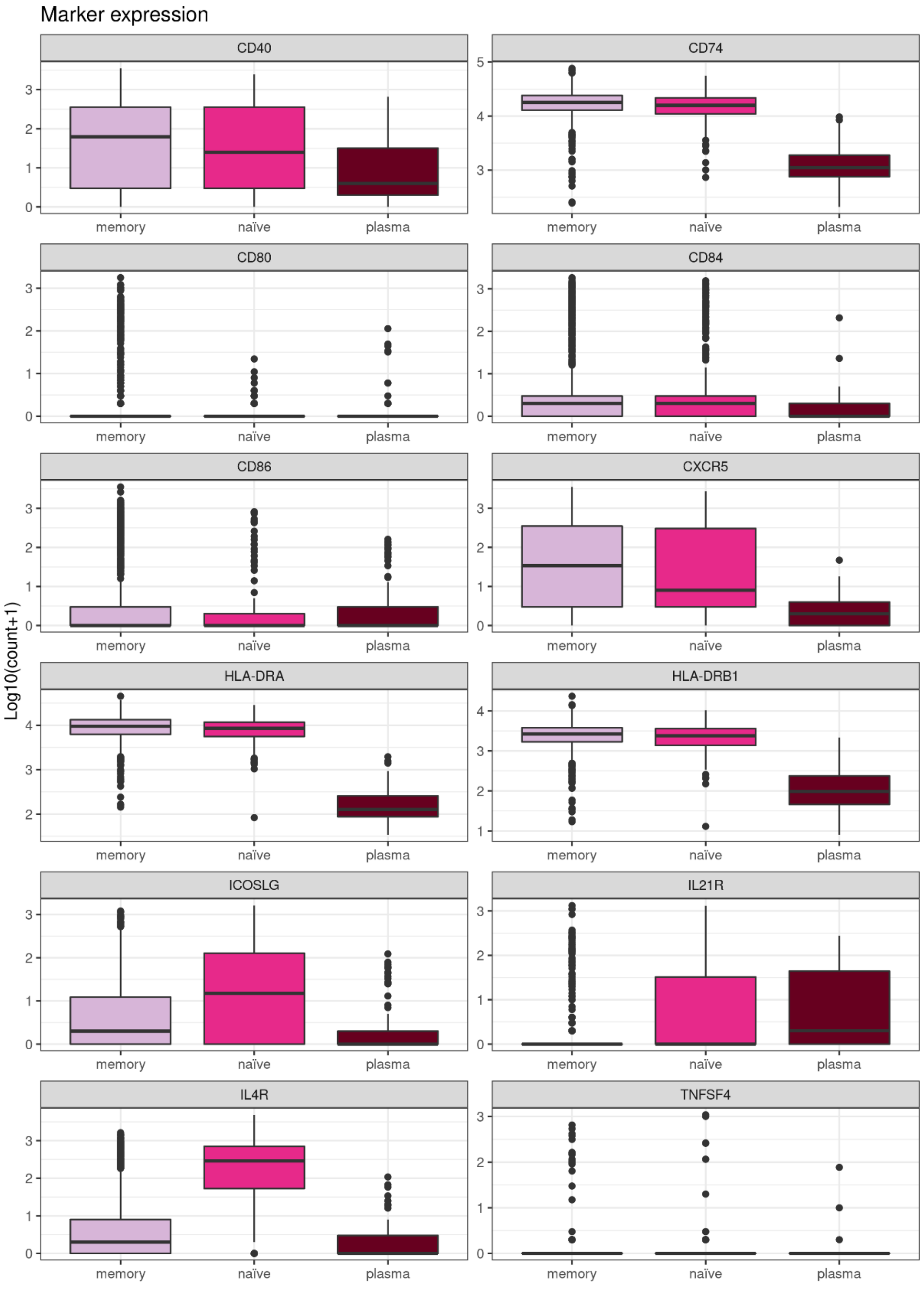

Fig. S7.

Logarithmic read count for B-T cell interaction markers are shown grouped by cell subset. Most markers are present in the naïve and the memory cell subset.

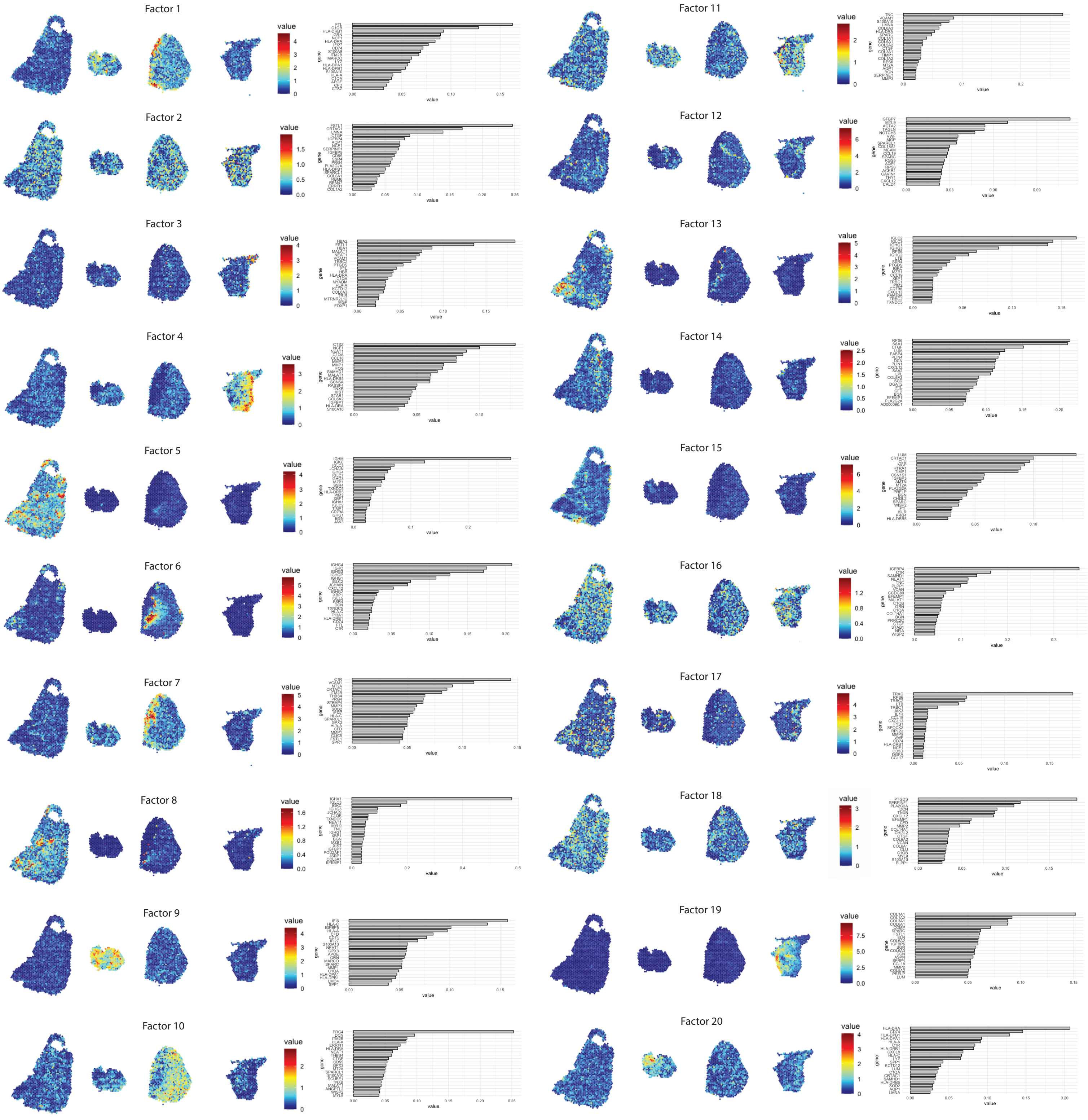
Factor 1
Factor 2
Factor 3
Factor 4
Factor 5
Factor 6
Factor 7
Factor 8
Factor 9
Factor 10
Factor 11
Factor 12
Factor 13
Factor 14
Factor 15
Factor 16
Factor 17
Factor 18
Factor 19
Factor 20
value

Fig. S8.

The factor analysis for spatial transcriptomics is shown. Most factors (e.g. 2,11,16,18) are represented on all tissues. Several factors have expression activities overlapping with distinct morphological areas such as infiltrates, connective tissue as well as areas bordering the infiltrates. These could be regarded as baseline tissue signals with *CD55* being of particular interest as it marks the intimal lining fibroblast-like synoviocytes. Three factors (6,8,13) shows a strong activity towards the immune cell-rich areas in biopsy A1. These areas correlate with IGH, IGL, *JCHAIN*, *MZB1*, *XBP1* and *CD79A* expression suggesting plasma cell presence. Factor 20 has strong activity within cluster 4 for biopsy A2. Here the driver genes are the plasma cell markers mentioned before as well as *HLA-DRB1* and *CD74* further highlighting antigen presentation. Biopsy A3 present factor 6 with strong activity in lymphocyte aggregates. Factor 6 is among others associated with the HLA-associated *CD74* gene. For biopsy A4, two of the factors (4,19) have genes associated with connective tissue and several HLA genes.

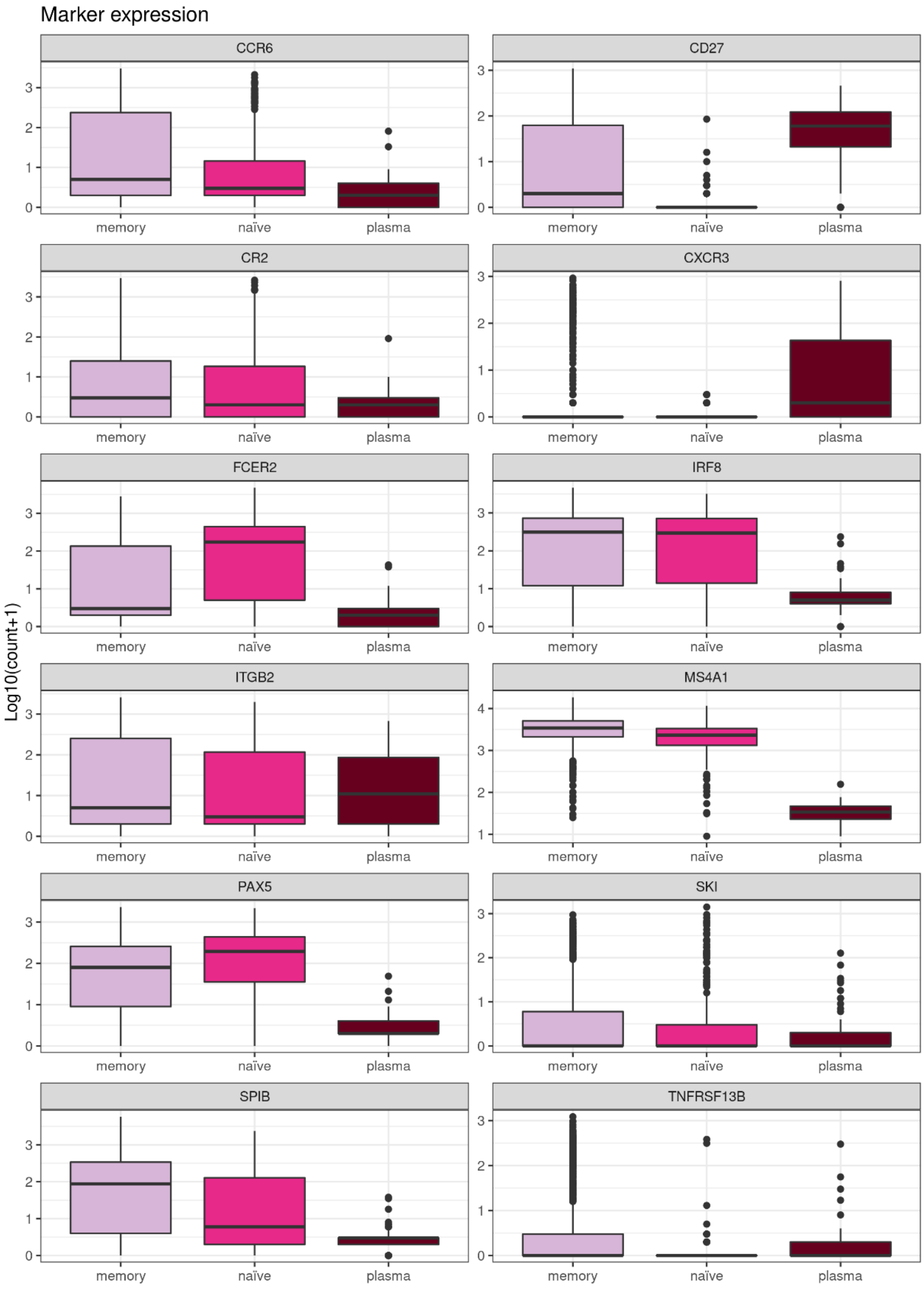

Fig. S9.

Logarithmic read count for memory B cell markers is shown grouped by cell subset. Most memory markers are either shared with the naïve or the plasma cell subset.

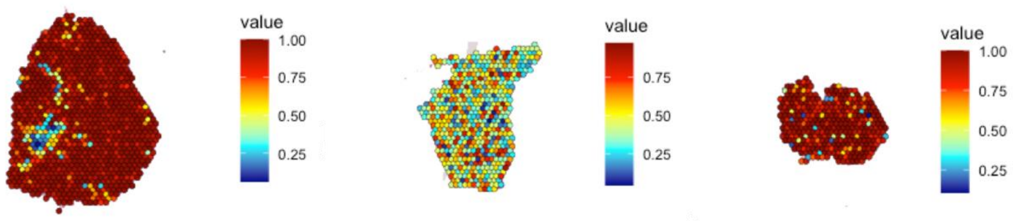

Fig. S10.

The memory B cell subset from paired data from the same individual was predicted on the tissue sections. The color bar represents the prediction score for memory B cells on the tissue sections.

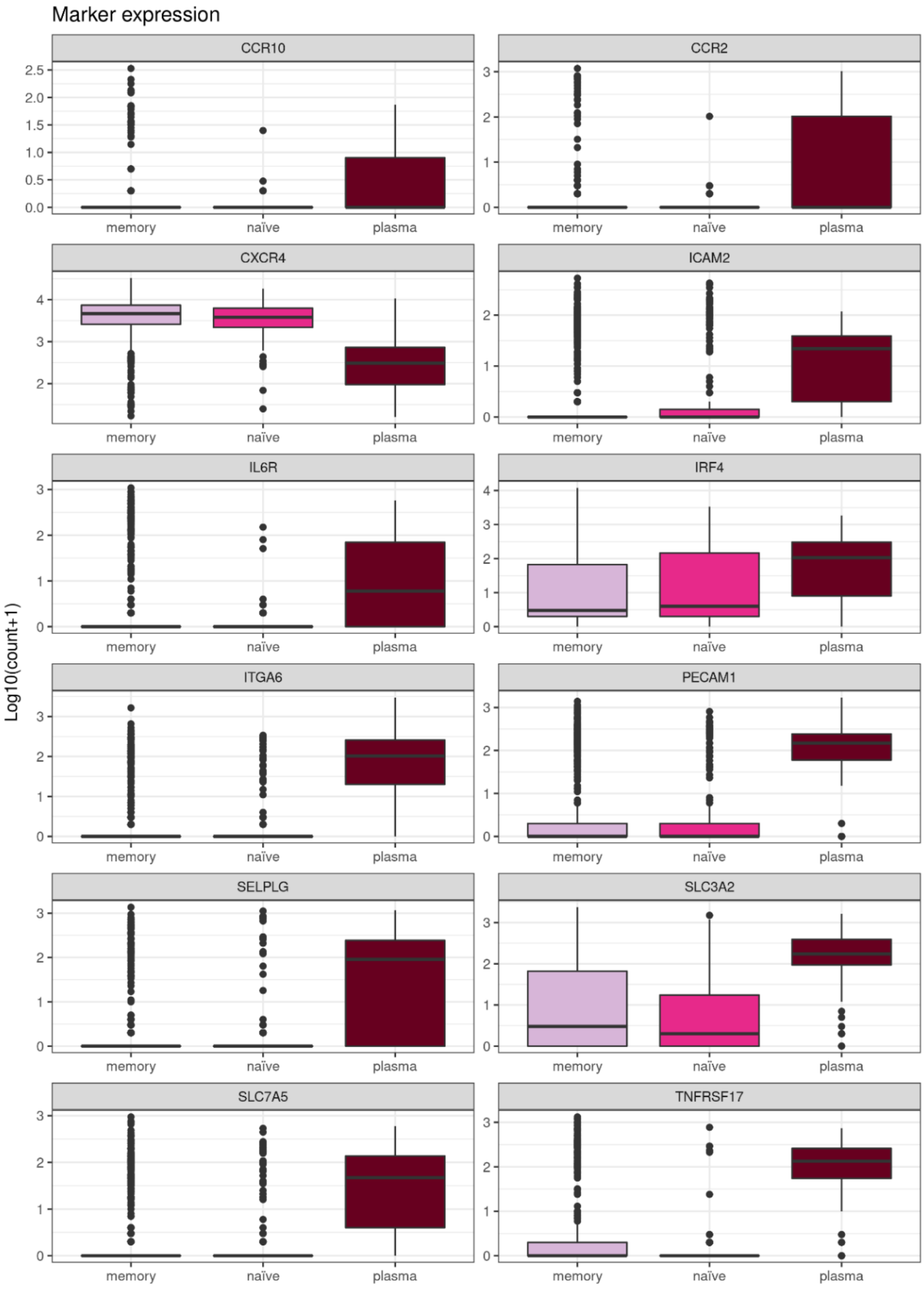

Fig. S11.

Logarithmic read count for plasma cell markers is shown grouped by cell subset. Most markers are clearly exclusive to plasma cells.

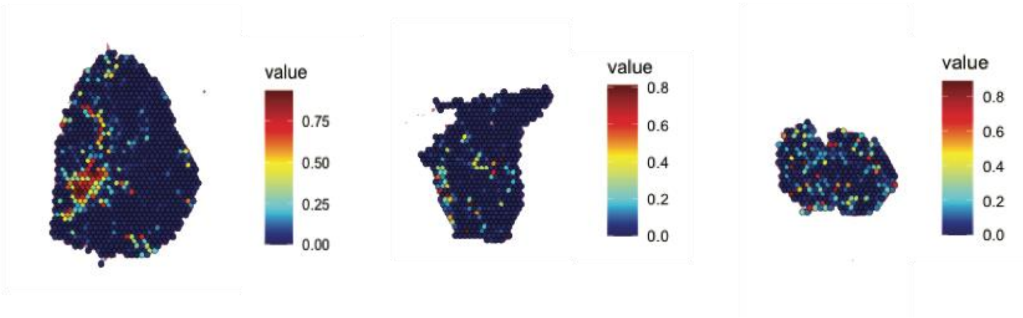

Fig. S12.

The plasma cell subset from paired data from one individual was predicted on the tissue sections. The color bar represents the prediction score for plasma cells on the tissue sections. Integration showed a clear correlation with the Zhang et al. [17] data.

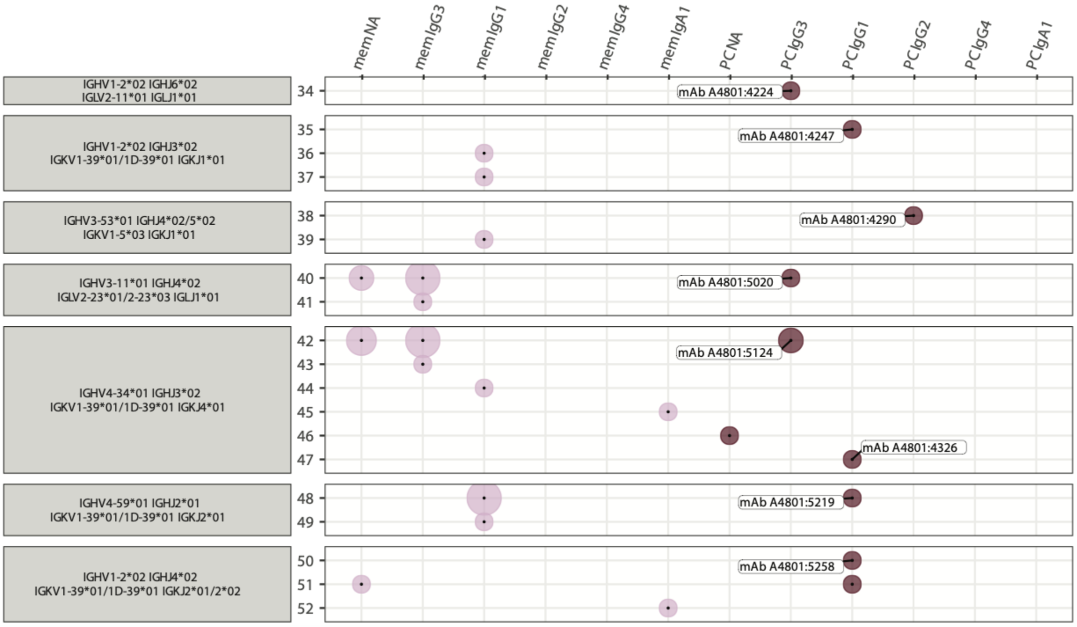

Clone	HCDR3	VH SHM (%)	LCDR3	VL SHM (%)
34	AGTNYDFWSGRPWYYYGMDV	1/294 (0.3)	CSYAGSYTYV	0/295 (0.0)
35	VRAARGSGWFHDVFDI	6/295 (2.0)	QQSYSTPRT	3/284 (1.1)
36	ARANFGGLRGYDAFDI	6/295 (2.0)	QQSYSTRT	3/283 (1.1)
37	ARANFGGLRGYDAFDI	8/295 (2.7)	QQSYSTRT	2/283 (0.7)
38	AKDGSSRSLVS	10/293 (3.4)	QQYNSFPWT	11/280 (3.9)
39	AKDGSSRSLVS	15/293 (5.1)	QQYNSYSPTWT	1/287 (0.3)
40	ARDKRRRHYDSSGYHFDY	6/296 (2.0)	CSYAGSSTSLYV	1/295 (0.3)
41	ARDKRRRHYDSSGYHFDY	6/295 (2.0)	CSYAGSSTSLYV	1/295 (0.3)
42	ARARSGISAIHGVFDI	3/293 (1.0)	QQSYSTPPT	4/287 (1.4)
43	ARARSGISAIHGVFDI	3/285 (1.1)	QQSYSTPPT	4/287 (1.4)
44	ARARSGLGATHGAFDI	7/292 (2.4)	QQSYSTPPT	1/287 (0.3)
45	ARGGGYGGNSVRRAFDI	28/293 (9.6)	QQTYITPRA	21/284 (7.4)
46	ARARSGLGATHGAFDI	4/292 (1.4)	QQSYSTPPT	4/287 (1.4)
47	ARARSGLGATHGAFDI	6/292 (2.1)	QQSFSTPPT	4/287 (1.4)
48	ARGKTGYSSRNHWYFDL	2/292 (0.7)	QQSSSTPMYT	2/284 (0.7)
49	ARGKTGYSSRNHWYFDL	2/292 (0.7)	QQSYSTPLST	2/284 (0.7)
50	ARGIMGQFPPDY	2/293 (0.7)	QQSYSTPQT	4/286 (1.4)
51	ARGIVGQFPPDH	8/293 (2.7)	QQSYSTPQT	2/286 (0.7)
52	AREVGVGAILDY	30/296 (10.1)	QQSYSTPYT	15/284 (5.3)

Fig. S13.

The plot shows the equivalent clonotype information that is shown in figure 6A for the seven plasma cell derived monoclonal antibodies originating from A3.

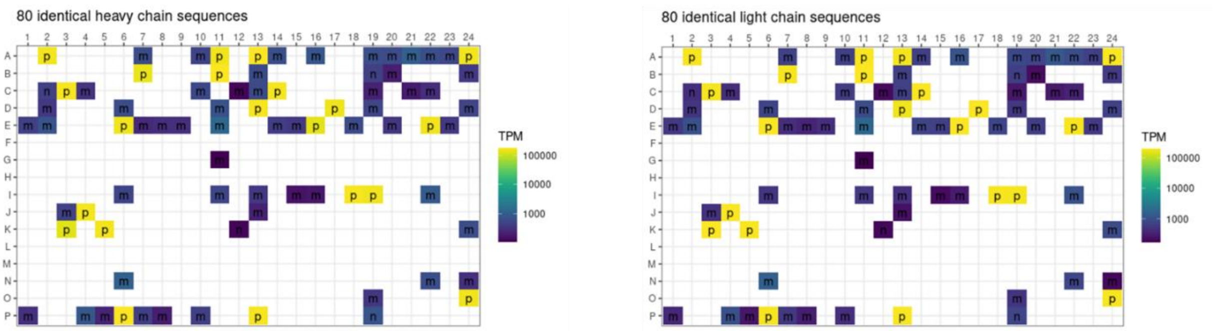

Fig. S14.

No index-hopping for monoclonal antibody A4797:2017. Cells expressing the A4797:2017 antibody were derived from plasma cells (p), memory B cells (m) and naïve B cells (n).

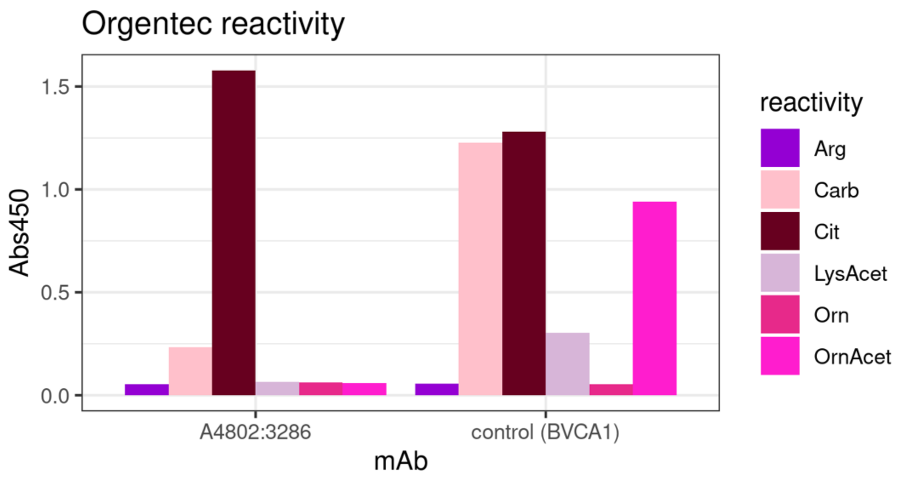

Fig. S15.

The Orgentec reactivity of the A4802:3286 clone compared to control (BVCA1 [22]) is shown. The ACPA clone was found to be negative for the acetylated peptide, weakly positive for the carbamylated peptide but strongly positive for the citrullinated peptide.

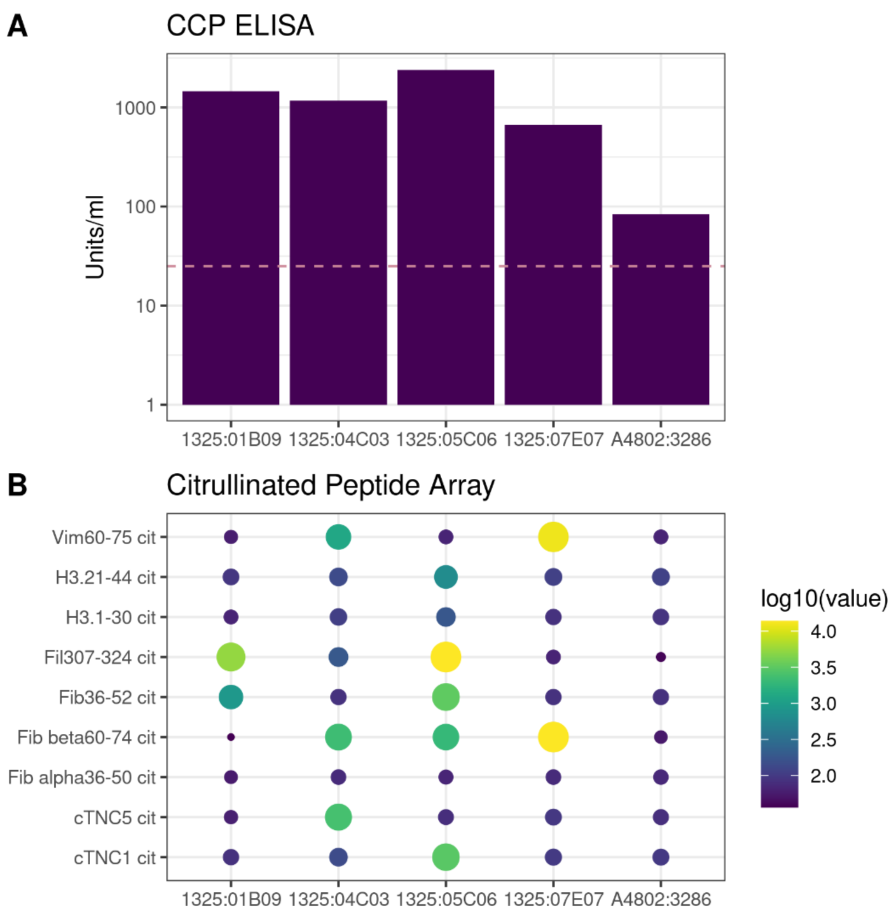

Fig. S16.

(A) CCP2 ELISA and **(B)** citrullinated peptide array [20] to test antigen specificity. We compared the identified A4802:3286 ACPA with previously described ACPAs generated from plasma cells from synovial fluid of RA patients with long lasting disease [40]. The cut-off for the anti-CCP2 ELISA was 25 units/ml. On the citrullinated peptide array, we assessed reactivity towards citrullinated Vimentin (Vim), citrullinated Histone 3 (H3), citrullinated Filaggrin (Fil), citrullinated Fibrinogen (Fib) and citrullinated Tenascin-C (TNC). Numbers indicate amino acid residues.

	N-X-S/T glycosylation sites			
mAb	germline VH	VH	germline VL	VL
A4797:2017	0	0	0	0
A4797:2216	0	0	0	0
A4801:4224	0	0	0	0
A4801:4247	0	1	0	0
A4801:4290	0	0	0	0
A4801:4326	2	2	0	0
A4801:5020	0	0	0	0
A4801:5124	1	1	0	0
A4801:5219	0	0	0	0
A4801:5258	0	0	0	0
A4802:3086	0	0	0	0
A4802:3286 (ACPA)	0	0	0	2
A4802:3314	0	0	0	0
A4802:6090	0	1	0	1
A4802:6117	0	0	0	0

Fig. S17.

Germline and germline-diverted glycosylation sites in monoclonal antibodies are shown.

Table S1.

cluster	gene	p_val	avg_logFC	pct_1	pct_2	p_val_adj
0	*MALAT1*	6.57E-69	-12.05	0.55	0.74	1.20E-64
0	*CD74*	1.63E-56	-60.97	0.82	0.90	2.97E-52
0	*EEF1A1*	9.36E-55	-21.51	0.74	0.84	1.70E-50
0	*MMP3*	3.91E-51	-136.45	0.60	0.75	7.11E-47
0	*ACTB*	1.67E-49	-19.56	0.80	0.88	3.05E-45
0	*B2M*	3.88E-48	-70.56	0.94	0.96	7.05E-44
0	*TMSB4X*	3.90E-48	-14.42	0.69	0.80	7.10E-44
0	*COL3A1*	6.07E-48	-73.30	0.63	0.77	1.10E-43
0	*FTH1*	1.73E-47	-23.46	0.67	0.79	3.15E-43
0	*HLA-B*	7.41E-47	-70.56	0.88	0.93	1.35E-42
1	*IGF1*	1.24E-77	0.97	0.21	0.06	2.26E-73
1	*COL1A1*	8.88E-52	190.30	0.77	0.59	1.62E-47
1	*MMP2*	1.77E-30	25.89	0.56	0.40	3.22E-26
1	*COL3A1*	5.12E-24	61.33	0.82	0.72	9.31E-20
1	*ELN*	1.59E-22	7.58	0.23	0.13	2.90E-18
1	*FNDC1*	3.89E-19	0.31	0.11	0.05	7.08E-15
1	*ASPN*	4.95E-19	6.42	0.12	0.06	9.02E-15
1	*AEBP1*	8.85E-19	21.02	0.36	0.25	1.61E-14
1	*COMP*	3.25E-18	2.98	0.23	0.14	5.92E-14
1	*CCL18*	3.77E-16	5.23	0.33	0.23	6.86E-12
2	*MMP3*	2.67E-276	118.78	0.94	0.69	4.86E-272

2	*MMP1*	1.04E-210	36.51	0.72	0.28	1.89E-206
2	*FN1*	9.72E-146	269.70	0.99	0.98	1.77E-141
2	*HTRA1*	9.61E-138	-22.09	0.88	0.72	1.75E-133
2	*PRG4*	7.54E-126	Inf	0.99	0.97	1.37E-121
2	*CRTAC1*	8.49E-125	18.19	0.85	0.67	1.54E-120
2	*MT2A*	3.02E-110	25.90	0.85	0.69	5.49E-106
2	*CLU*	5.26E-110	-76.09	0.95	0.88	9.57E-106
2	*VCAM1*	9.61E-102	1.96	0.76	0.53	1.75E-97
2	*PLA2G2A*	5.94E-97	8.88	0.81	0.62	1.08E-92
3	*MALAT1*	1.21E-125	-4.57	1.00	0.67	2.21E-121
3	*ACTB*	8.96E-66	-34.59	0.77	0.88	1.63E-61
3	*EEF1A1*	6.41E-65	-23.05	0.71	0.83	1.17E-60
3	*HLA-B*	1.12E-58	-108.00	0.87	0.93	2.03E-54
3	*RPL13A*	4.02E-58	-12.76	0.46	0.68	7.31E-54
3	*TMSB4X*	3.17E-57	-27.81	0.65	0.79	5.77E-53
3	*MT-CO3*	5.39E-57	-53.99	0.86	0.92	9.81E-53
3	*TMSB10*	1.31E-56	-23.63	0.57	0.74	2.38E-52
3	*MT-ND1*	5.70E-56	-20.54	0.71	0.84	1.04E-51
3	*MT-ND4*	2.13E-55	-32.26	0.75	0.86	3.87E-51
4	*JCHAIN*	7.99E-184	42.19	0.78	0.35	1.45E-179
4	*IGH*	2.73E-141	Inf	0.97	0.84	4.97E-137
4	*MZB1*	6.11E-97	2.10	0.49	0.19	1.11E-92
4	*IGHGP*	2.00E-92	74.42	0.42	0.15	3.63E-88

4	*TXNDC5*	5.11E-77	9.17	0.57	0.29	9.30E-73
4	*DERL3*	4.95E-72	0.67	0.26	0.07	9.02E-68
4	*CD79A*	4.75E-58	0.57	0.24	0.07	8.64E-54
4	*XBP1*	4.74E-57	4.20	0.47	0.24	8.62E-53
4	*PIM2*	1.03E-52	0.93	0.25	0.08	1.88E-48
4	*SDC1*	1.82E-50	0.93	0.23	0.07	3.31E-46
5	*HLA-DQA1*	5.13E-72	1.70	0.62	0.34	9.34E-68
5	*LYZ*	3.39E-66	4.87	0.68	0.42	6.17E-62
5	*CD74*	3.22E-56	2.16	0.94	0.88	5.86E-52
5	*HLA-DRA*	6.95E-44	31.21	0.90	0.87	1.26E-39
5	*CXCL9*	3.52E-41	2.41	0.41	0.21	6.41E-37
5	*HLA-DRB1*	2.76E-38	5.17	0.78	0.67	5.02E-34
5	*FTH1*	3.77E-37	1.21	0.84	0.76	6.86E-33
5	*FTL*	1.95E-36	2.99	0.90	0.86	3.55E-32
5	*HLA-DPA1*	2.04E-36	6.94	0.75	0.60	3.71E-32
5	*TMSB10*	4.28E-36	11.04	0.82	0.71	7.79E-32
6	*IGFBP7*	1.01E-164	2.96	0.82	0.40	1.84E-160
6	*ACTA2*	1.87E-148	4.21	0.48	0.12	3.40E-144
6	*TAGLN*	1.90E-112	1.93	0.42	0.12	3.46E-108
6	*MCAM*	7.17E-101	0.81	0.33	0.08	1.30E-96
6	*VWF*	1.60E-86	2.41	0.35	0.10	2.91E-82
6	*MYL9*	1.06E-73	2.30	0.64	0.34	1.92E-69
6	*NR2F2*	5.89E-58	0.61	0.20	0.05	1.07E-53

6	*ACKR1*	4.38E-49	0.64	0.19	0.05	7.97E-45
6	*CCL19*	4.31E-48	17.11	0.29	0.11	7.85E-44
6	*NOTCH3*	1.16E-40	0.71	0.30	0.12	2.12E-36
7	*PRELP*	9.81E-63	5.75	0.56	0.17	1.78E-58
7	*HTRA1*	2.30E-53	27.27	0.96	0.73	4.18E-49
7	*PRG4*	3.43E-26	-Inf	0.99	0.97	6.24E-22
7	*CSN1S1*	2.43E-22	5.88	0.25	0.08	4.43E-18
7	*FN1*	2.58E-22	-264.68	0.99	0.98	4.70E-18
7	*AMTN*	2.65E-22	10.23	0.21	0.06	4.82E-18
7	*CRTAC1*	1.36E-21	-13.01	0.80	0.69	2.48E-17
7	*CLU*	8.46E-20	81.27	0.90	0.89	1.54E-15
7	*IGFBP5*	1.34E-19	-2.67	0.67	0.46	2.44E-15
7	*SLC39A14*	1.70E-19	-0.47	0.47	0.24	3.10E-15
8	*FABP4*	1.51E-47	5.49	0.26	0.05	2.75E-43
8	*PLIN4*	1.03E-41	0.42	0.19	0.03	1.88E-37
8	*LPL*	2.04E-37	0.79	0.15	0.02	3.71E-33
8	*C3*	1.15E-33	-0.51	0.59	0.27	2.09E-29
8	*SCD*	1.48E-23	0.74	0.26	0.08	2.70E-19
8	*SAA1*	6.65E-19	0.86	0.18	0.05	1.21E-14
8	*B2M*	4.73E-12	-155.66	0.96	0.96	8.60E-08
8	*HLA-A*	8.23E-11	-65.66	0.69	0.80	1.50E-06
8	*HLA-B*	1.13E-10	-119.66	0.91	0.92	2.05E-06
8	*HLA-E*	6.72E-10	-4.90	0.56	0.68	1.22E-05

9	*HBA2*	0.00E+00	2.00	1.00	0.05	0.00E+00
9	*HBA1*	7.79E-33	1.84	0.14	0.02	1.42E-28
9	*MMP2*	1.82E-08	-26.12	0.24	0.43	3.32E-04
9	*CLU*	3.92E-08	-149.47	0.78	0.89	7.14E-04
9	*CD68*	8.59E-08	-4.06	0.19	0.36	1.56E-03
9	*ENO1*	1.02E-07	-4.21	0.26	0.44	1.86E-03
9	*PSAP*	1.39E-07	-24.50	0.72	0.81	2.53E-03
9	*SAT1*	1.73E-07	-4.67	0.29	0.46	3.16E-03
9	*CTSB*	2.22E-07	-19.17	0.67	0.77	4.04E-03
9	*HTRA1*	2.70E-07	-96.38	0.66	0.74	4.91E-03

Top 10 differentially expressed genes for 10 spatial transcriptomics clusters are shown.

Table S2.

	OR	p-value
Naïve B cells (SC-B1)	20.3	<2.2e-16
Memory B cells (SC-B2)	99.6	<2.2e-16
Plasma cells (SC-B4)	3.27	<2.2e-16

Fisher’s exact test for memory B cells and the naïve B cells, memory B cells and plasma cells clusters from previously published studies [17] is shown.

Table S3.

	OR	p-value
Naïve B cells (SC-B1)	0.438	1
Memory B cells (SC-B2)	0	1
Plasma cells (SC-B4)	45.1	<2.2e-16

Fisher's exact test for plasma cells and the naïve B cells, memory B cells and plasma cell clusters from previously published studies [17] is shown.
